# A Workflow for Selecting, Profiling, and Optimizing Plant Extracts for Cosmetic Applications

**DOI:** 10.1002/cbdv.202502397

**Published:** 2025-10-22

**Authors:** Maria Viéytez, Aline Robert‐Hazotte, Amélie Thomas, Véronique Nardello‐Rataj, Xavier Fernandez

**Affiliations:** ^1^ Institut De Chimie de Nice Université Côte d'Azur Nice France; ^2^ Shiseido Europe Innovation Center Ormes France; ^3^ Futura Gaia Technologies Rodilhan France; ^4^ Unité De Catalyse et Chimie du Solide Centrale Lille, Université de Lille, Université Artois Lille France

**Keywords:** antioxidant activity, cosmetic bioassay, natural cosmetic ingredient, phytocosmetics, rotational cultivation

## Abstract

In response to the growing demand for innovative natural ingredients in cosmetics, this study proposes a structured approach to identifying bioactive plant species available in France. From 1614 species screened for regulatory acceptability and innovation potential, 18 were shortlisted for phytochemical profiling (high‐performance liquid chromatography [HPLC]–diode array detector [DAD]/evaporative light scattering detector [ELSD]) and in vitro antioxidant and enzymatic assays. A dual scoring system combining bioactivity and novelty metrics highlighted four promising species for cosmetic development. To explore how cultivation methods can optimize ingredient performance, *Borago officinalis* L., a fifth species with moderate initial scores, was selected for a case study based on its agronomic feasibility, identified with an industrial partner. Cultivated indoors under controlled conditions using a rotational geoponics system, borage showed improved antioxidant properties under low‐temperature conditions. These findings provide a replicable workflow for sourcing plant‐based natural active ingredients and illustrate how tailored cultivation can unlock the potential of well‐known but underexploited species.

## Graphical abstract

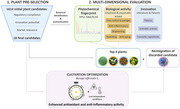

## 1 Introduction

The cosmetics industry faces a unique set of challenges in the development of new active ingredients, as it must meet strict regulatory requirements, demonstrate safety and efficacy, and respond to evolving consumer expectations. Recent shifts in consumer behavior have sparked growing interest in cosmetic products that include natural ingredients [1]. Although synthetic ingredients remain essential for many formulations, an increasing number of consumers are seeking formulas that incorporate natural and sustainably sourced components, which they perceive to be safer, more environmentally friendly, and aligned with their values.

At the same time, consumers remain highly demanding in terms of product performance [2]. A high level of efficacy is now expected, as individuals seek clear, demonstrable benefits that justify their cosmetic expenditures. This dual expectation for both natural origin and measurable effectiveness has significantly raised the bar for the development of new cosmetic actives.

In this context, plants have become an essential source for developing natural active ingredients in skincare, offering solutions for needs such as hydration, sun protection, anti‐aging, and skin brightening [1]. With approximately 500 million years of evolution, plants have adapted to survive extreme conditions like UV radiation, fires, and ice ages. Today, there are about 350 000 known plant species, each producing compounds with diverse chemical structures and biological activities that play crucial roles in survival and reproduction [3]. These natural mechanisms, from hydration to protection against environmental stressors, provide valuable inspiration for creating active ingredients that meet both consumer expectations and industry standards.

However, the process of integrating plant‐based compounds into cosmetic formulations involves navigating numerous constraints specific to this sector. Beyond scientific validation and economic feasibility, ingredient selection must consider formulation compatibility, supply chain sustainability, and strict regulatory compliance across international markets [4]. This study focuses on the early stages of developing innovative plant‐based active ingredients for skincare, with a particular emphasis on sourcing from the botanical diversity available in mainland France (excluding overseas territories). Rather than limiting the scope to endemic or native species, the selection was based on plants that are present, accessible, and potentially cultivable within this geographic area. This definition of “local” sourcing was chosen to align with industrial feasibility, sustainability goals, and traceability requirements of this work. The plant selection process described herein was also designed to comply with global cosmetic regulations, with the objective of identifying ingredients that could be successfully developed and commercialized worldwide.

A strategic screening process has been implemented to effectively navigate these challenges and identify the most promising plant candidates accessible in France. The main steps of the screening methodology adopted are discussed, and the meticulous selection of plants and their study is explained, with the aim of identifying those with the highest potential for further innovative developments. Untargeted analysis of the phytochemical profile, combined with the assessment of the biological activity using chemical and enzymatic assays, allowed the selection of candidate plants to be narrowed down. By establishing a robust foundation for screening several plants locally sourced in France, this work aims to meet the objectives of the early stages of the development of innovative active ingredients based on French flora.

## 2 Results and Discussion

### 2.1 Strategy for the Selection of Candidate Plants

**FIGURE 1:**
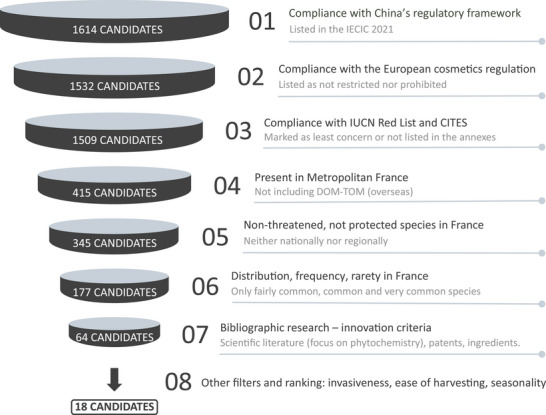
Summary diagram of the funnel strategy for selecting plants. Numbers show the remaining candidates after each filtering step, starting from 1614 species authorized within China's cosmetic framework.

To identify plants with potential for innovative cosmetic applications, a systematic selection process was employed, integrating key criteria such as regulatory compliance, biodiversity conservation, sourcing feasibility, and innovation potential. This process followed a funnel strategy (Figure 1), progressively refining a broad initial list to a final selection of 18 species for in‐depth study.

The starting point was a pool of 1614 candidate plants, identified on the basis of their authorization for cosmetic use in the Chinese regulatory framework. This selection criterion was prioritized due to the stringent nature of Chinese cosmetic regulations, which are among the strictest globally. The Inventory of Existing Cosmetic Ingredients in China (IECIC 2021) serves as a positive list, meaning only listed ingredients are permitted in cosmetics commercialized in China [5, 6]. Ensuring compliance with this framework from the very beginning enhances, but does not ensure, the potential for global market integration, as Chinese‐approved ingredients are more likely to meet or exceed regulatory requirements in other regions. This “worldwide” approach is particularly appealing to global cosmetic companies, as it enables seamless integration of the same ingredient across various markets, avoiding the need for extensive modifications according to the region of commercialization and thus reducing the need for region‐specific formulations and streamlining commercialization. In contrast, selecting plants that are only authorized in certain areas may restrict international distribution, complicating marketing strategies and reducing commercialization efficiency. Moreover, prioritizing ingredients authorized in China is not only a regulatory safeguard but also a strategic business decision, as China represents a major market for cosmetic innovation, particularly for premium and luxury brands.

Plant‐derived ingredients were identified from the IECIC list, filtering out non‐botanical materials, including isolated molecules and synthetic compounds, and retaining only botanical materials, such as extracts, oils, and powders. This step resulted in an initial selection of 1614 unique plant candidates.

The selection was further refined by applying the European Cosmetic Regulation, which operates with a negative list for prohibited and restricted substances and positive lists for allowed dyes, preservatives, and UV filters [7]. Plants explicitly prohibited or subject to restrictions in Europe were excluded, resulting in a reduced list of 1532 potential candidates. This step ensured that candidates authorized for cosmetic use in China but banned or restricted in Europe were removed. Although relatively few such cases exist, some examples persist. For instance, *Tagetes erecta* flower extract is permitted in China but has been prohibited in the EU since 2019 due to the absence of an established safe concentration for cosmetic use [5, 8].

To ensure the protection of biodiversity, conservation laws and endangered species lists were taken into account. The Red List of the International Union for Conservation of Nature (IUCN) and the annexes of the Convention on International Trade in Endangered Species of Wild Fauna and Flora (CITES) were consulted [9, 10, 11]. These resources provide critical information on species at risk of extinction or subject to legal protection in at least one country. By considering all three CITES annexes, species with potential extinction risks were proactively excluded, reinforcing the sustainability of the selected candidates. This precautionary approach not only mitigates future conservation concerns but also safeguards the reputation of potential cosmetic ingredients by avoiding species that are protected in sourcing regions. As a result, the list was further refined to 1509 species.

Given the potential for local ingredient sourcing and the botanical richness of the French territory, the selection was further refined to focus on plant species present in mainland France, excluding overseas territories [12]. France encompasses a wide range of climates, including Mediterranean, oceanic, continental, and alpine influences, supporting a diverse flora with unique bioactive properties. Prioritizing species that are locally available and potentially cultivable contributes to sustainability and supply chain resilience. Applying this criterion reduced the list of candidates to 415 species.

Focusing on French botanical resources, additional conservation databases were consulted to ensure the sustainability of selected species. The National Inventory of Natural Heritage (INPN) was used to identify plants classified as threatened in at least one region of France [12, 13]. Protected species, often subject to restrictions on wild harvesting, were excluded to prevent sourcing challenges and potential future regulatory bans. This step maintained the list at 415 species. To further refine the selection, species distribution data from the same databases were analyzed. Preference was given to plants categorized as common to very common in France, ensuring accessibility and alignment with the notion of valorizing widely recognized native flora. Conversely, rare to extremely rare species were excluded, reducing the list to 177 candidate plants [12, 13].

Following the selection of 177 candidate plants, a comprehensive bibliographic review was conducted to assess their potential for innovative cosmetic applications. This review aimed to evaluate traditional uses, biological activities, phytochemical composition, and existing industrial applications to identify the most promising species for further study.

To ensure a thorough evaluation, multiple databases were consulted:

- Scientific literature: CAS SciFinder, Science Direct, PubMed, and Google Scholar were used to collect data on traditional uses, biological activities relevant to cosmetics and pharmaceuticals, phytochemical composition, and any reported toxicological concerns. Particular attention was given to identified bioactive compounds and their documented effects.
- Patent databases: WIPO, Espacenet, and Google Patents were searched to identify existing patents related to the cosmetic use of the candidate plants.
- Cosmetic ingredient databases: UL Prospector, SpecialChem, PCPC Database, INCI Beauty, INCIDecoder, and COSMILE Europe were examined to assess the presence of the plants in marketed cosmetic formulations. This analysis focused on their prior use, extract types, reported bioactivities, and associated brands. Additionally, the patent status of relevant ingredients was verified to evaluate their commercial potential.

The selection process prioritized species with limited prior study, minimal market presence, and underexplored bioactive potential. However, it is important to recognize that compliance with Chinese cosmetic regulations requires a substantial body of existing documentation, including phytochemical and toxicological data. Consequently, focusing on plants already approved under Chinese regulations inherently favors relatively well‐documented species, which may introduce a bias in the innovative aspect of this work. Although this approach ensures regulatory feasibility and data availability, it may underestimate the novelty of less‐documented species that could also hold promising cosmetic potential. Despite this constraint, the bibliographic review identified 64 candidate plants that, although compliant with Chinese regulatory standards, remain relatively underutilized in the cosmetics sector.

The final stage of the selection process considered the feasibility of industrial development for plant‐derived ingredients. Beyond regulatory and ecological criteria, practical aspects such as the existence of an established supply chain or the ease of implementing one were evaluated. This step ensured that selected plants could be sustainably sourced at an industrial scale without major logistical constraints. Additionally, in alignment with responsible sourcing practices, invasive species were excluded at this stage to prevent potential ecological disruptions and support biodiversity conservation. By integrating these considerations, a final shortlist of 18 candidate plants was established (Table 1). These species meet all previously defined criteria, including compliance with Chinese and European cosmetic regulations, adherence to biodiversity protection frameworks, presence in continental France, and high innovation potential for cosmetic applications.

**TABLE 1:** Candidate plants resulting from the selection process.

Family	Botanical name	Common name	Plant parts	Chemical composition and bioactivity mentions	References
Amaryllidaceae	*Narcissus jonquilla* L.	Jonquil	Aerial parts	Contains alkaloids; limited data on cosmetic applications outside of perfumery; primarily ornamental use	[14, 15]
Asparagaceae	*Polygonatum multiflorum* (L.) All	Solomon's‐seal	Roots/Rhizome	Contains saponins and flavonoids; very limited data concerning cosmetic use	[16]
Asteraceae	*Achillea millefolium* L.	Common yarrow	Aerial parts	Contains sesquiterpenes, flavonoids, and phenolic acids; wound‐healing, anti‐inflammatory, antimicrobial, and antioxidant properties	[17, 18]
	*Lactuca serriola* L.	Prickly lettuce	Leaves	Limited research on cosmetic applications; some antioxidant activity	[19]
Berberidaceae	*Berberis aquifolium* Pursh	Holly‐leaved barberry	Aerial parts	Contains alkaloids; used to treat skin complaints (dryness, psoriasis), anti‐bacterial, and antioxidant properties	[20, 21]
Betulaceae	*Betula pendula* Roth	Silver birch	Aerial parts	Contains terpenes, flavonoids, catechins; wound‐healing, anti‐inflammatory, antimicrobial, and antioxidant properties	[22]
Betulaceae	*Corylus avellana* L.	Common hazel	Leaves	Contains phenolic compounds; antioxidant properties	[23, 24]
Boraginaceae	*Borago officinalis* L.	Borage	Aerial parts	Contains phenolic acids, flavonoids, and fatty acids; anti‐inflammatory, antibacterial, and antioxidant properties	[25]
Brassicaceae	*Capsella bursa‐pastoris* (L.) Medik	Shepherd's purse	Aerial parts	Contains flavonoids, fatty acids, phenolic acids; wound‐healing, anti‐inflammatory, and antioxidant properties	[26, 27]
Caprifoliaceae	*Dipsacus fullonum* L.	Fuller's teasel	Aerial parts and roots	Limited research, but contains iridoids and phenolic acid; some antioxidant and anti‐inflammatory activity reported	[28]
Fabaceae	*Spartium junceum* L.	Spanish broom	Flowers	Contains flavonoids; anti‐inflammatory and antioxidant properties	[29]
Portulacaceae	*Portulaca oleracea* L.	Purslane	Aerial parts and roots	Contains flavonoids, alkaloids, fatty acids, terpenoids, sterols; anti‐inflammatory, wound‐healing, and antioxidant properties	[30]
Primulaceae	*Primula veris* L.	Cowslip primrose	Aerial parts	Contains phenolic glycosides, saponins, flavonoids, and phenolic acids; anti‐inflammatory, antibacterial, and antioxidant properties	[31, 32]
Plantaginaceae	*Plantago lanceolata* L.	Ribwort plantain	Aerial parts	Contains iridoid glucosides and phenylethanoid glycosides; wound‐healing and anti‐inflammatory	[33]
	*Plantago major* L.	Broadleaf plantain	Aerial parts	Contains polysaccharides, lipids, caffeic acid derivatives, flavonoids, iridoid glycosides, and terpenoids; wound‐healing, anti‐inflammatory, and antioxidant properties	[34]
Rosaceae	*Agrimonia eupatoria* L.	Agrimony	Aerial parts	Contains tannins, flavonoids, phenolic acids, and triterpenoids; anti‐inflammatory and antioxidant properties	[35, 36]
	*Cormus domestica* (L.) Spach	Service tree	Buds	Contains phenolic compounds with potential antioxidant activity	[37]
Ranunculaceae	*Clematis vitalba* L.	Old‐man's‐beard	Leaves	Contains saponins, sterols, lactones; anti‐inflammatory properties	[38]

All 18 harvested plants were systematically identified at the time of collection by an expert botanist (see Section 4). This step is essential to ensure that the correct species are studied, to guarantee the reliability of the subsequent analyses, and to support transparency in the sourcing of botanical materials.

### 2.2 Preliminary Study of Candidate Plants

**FIGURE 2:**
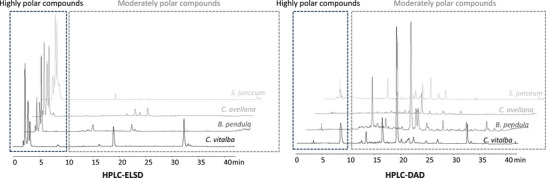
HPLC–DAD/ELSD chromatogram of four different plant species. DAD, diode array detector; ELSD, evaporative light scattering detector; HPLC, high‐performance liquid chromatography.

To assess the potential of each selected plant, extractions were performed under standardized conditions (details in Section 4). Briefly, two types of extracts were obtained for each plant: a dry hydroalcoholic extract and a liquid propylene glycol extract. This approach ensured a broad coverage of extracted metabolites, yielding extracts with potentially diverse bioactivities. The dry extracts (DEs) were then analyzed using high‐performance liquid chromatography (HPLC)–diode array detector (DAD)/evaporative light scattering detector (ELSD) to generate phytochemical fingerprints, facilitating comparative metabolomic profiling. This untargeted analysis allowed for the selection of the most promising candidates under equivalent extraction and analytical conditions. At this stage, the study aimed at obtaining a broad phytochemical fingerprint rather than detailed structural elucidation. Although HPLC–DAD/ELSD profiling provided useful comparative fingerprints, it does not allow the structural elucidation of the metabolites responsible for the observed bioactivities. As such, the approach was sufficient for rapid screening and prioritization, but future applications of this workflow should incorporate complementary techniques such as LC–MS and bio‐guided fractionation to enable compound‐level identification and strengthen the link between phytochemical profiles and biological effects. Representative chromatographic profiles of *Spartium junceum*, *Corylus avellana*, *Betula pendula*, and *Clematis vitalba* are presented in Figure 2 to illustrate an example of the variability between the obtained extracts, but the remaining chromatograms are available in the Supporting Information section.

Following this untargeted chromatographic profiling, priority was given to species with diverse chemical profiles and/or those containing high concentrations of moderately polar compounds. These compounds, deduced from their retention times and detection in HPLC–DAD/ELSD, include phenolic compounds (such as flavonoids, lignans, stilbenes, and phenolic acids) and terpenes/terpenoids, which are of interest due to their potential bioactivity and relevance for cosmetic applications. On the basis of the comparative chromatographic profiles shown in Figure 2, representing only a subset of the analyzed species, *C. vitalba* stood out due to its particularly rich and varied chromatographic profile, including two major moderately polar compounds that may be linked to a certain bioactivity.

Although chromatographic untargeted profiling provides valuable insights into the chemical diversity of the extracts, it does not directly result in precise compound identification or biological efficacy. To further evaluate their potential for cosmetic use, a series of chemical and enzymatic in vitro assays was conducted. Antioxidant activity was assessed using the DPPH radical scavenging assay, whereas enzymatic inhibition assays targeted key skin‐related enzymes: hyaluronidase (hydration), tyrosinase (skin brightening), lipoxygenase (anti‐inflammatory), and collagenase (anti‐aging) [39]. All results are based on three technical replicates (*N* = 3) per extract; no independent biological replicates were performed, so trends reported should be interpreted as indicative rather than definitive. Future studies, including independent plant collections, will be important to confirm the robustness of these findings. Results are summarized in Table 2, with percentage inhibition values and antioxidant capacity. For reference, each assay included two positive controls: a commercial plant extract, marketed for the targeted activities, and a pure molecular reference, selected on the basis of well‐documented activity in the literature (e.g., kojic acid for anti‐tyrosinase activity), allowing comparison of the plant extracts with established benchmarks. For easier cross‐species comparison, inhibition values were also converted to a semi‐quantitative format using a “+” scale, and a standardized bioactivity score (0–100) was calculated and averaged per species (see Section 4 for details).

**TABLE 2:** Biological activities of the extracts.

Botanical name	Extract	Bioactivity					Bioactivity score per extract out of 100	Average bioactivity score per plant out of 100
		DPPH	Anti‐hyaluronidase	Anti‐tyrosinase	Anti‐lipoxygenase	Anti‐collagenase		
Commercial reference		++++	++++	++++	++++	+	80	
Molecular reference		++++	++++	++++	++++	+++	85	
*Achillea millefolium*	HA	++++	++++	−	−	−	40	38
	PG	+++	++++	−	−	−	35	
*Agrimonia eupatoria*	HA	++++	++++	−	+++	−	55	53
	PG	++++	++++	−	++	−	50	
*Berberis aquifolium*	HA	++++	n.d.	−	++	−	37.5	41
	PG	++++	n.d.	−	+++	−	43.8	
*Betula pendula*	HA	+++	++++	−	+++	−	50	55
	PG	++++	++++	−	++++	−	60	
*Borago officinalis*	HA	++	++	−	+	−	25	30
	PG	++	++++	−	+	−	35	
*Capsella bursa‐pastoris*	HA	−	n.d.	−	++	−	12.5	22
	PG	+	n.d.	−	++++	−	31.3	
*Clematis vitalba*	HA	++++	++++	−	+++	−	55	60
	PG	++++	++++	+	+++	+	65	
*Cormus domestica*	HA	++++	n.d.	++	+	n.d.	58.3	54
	PG	++	n.d.	++	++	n.d.	50	
*Corylus avellana*	HA	+++	++++	−	++++	−	60	60
	PG	++++	++++	−	++++	−	60	
*Dipsacus fullonum*	HA	++	++++	−	++	n.d.	50	50
*Lactuca serriola*	HA	++++	+++	n.d.	+	n.d.	66.7	54
	PG	++	+++	n.d.	−	n.d.	41.7	
*Narcissus jonquilla*	HA	+	+	−	+	n.d.	18.8	12
	PG	−	+	−	−	n.d.	5	
*Plantago lanceolata*	HA	+	++++	−	−	−	25	30
	PG	+	++++	−	−	++	35	
*Plantago major*	HA	++++	++++	−	+	−	45	43
	PG	++++	++++	−	−	−	40	
*Polygonatum multiflorum*	HA	−	n.d.	+	−	n.d.	8.3	4
	PG	−	n.d.	−	−	n.d.	0	
*Portulaca oleracea*	HA	−	+	−	−	−	5	13
	PG	−	++++	−	−	−	20	
*Primula veris*	HA	+	++++	−	++	−	35	40
	PG	+++	++++	−	++	−	45	
*Spartium junceum*	HA	+	+	n.d.	−	n.d.	16.7	25
	PG	++	++	n.d.	−	n.d.	33.3	

*Note*: “−”: Inhibition < 20%; “+”: 20% < inhibition < 40%; “++”: 40% < inhibition < 60%; “+++”: 60% < inhibition < 80%; “++++”: inhibition > 80%; n.d.: not determined.

Abbreviations: HA, hydroalcoholic extract; PG, propylene glycol.

For this study, antioxidant capacity was assessed using only the DPPH chemical assay, which does not fully reflect biological relevance. This methodology could be strengthened by including additional complementary antioxidant assays to provide a more rigorous assessment. Similarly, enzyme inhibition was assessed at a single concentration (100 µg/mL per well) for all extracts to allow rapid comparative screening of a large number of species in a single 96‐well plate. Although this approach allows for prioritization, it does not provide IC_50_ values. Future studies should incorporate additional in vitro tests and cell models and determine IC_50_ values for the most promising extracts to confirm their efficacy and rule out any potential cytotoxicity.

At this stage, bio‐guided fractionation could be envisaged to identify the compounds responsible for the observed activities in each extract. However, the aim of the present work was not an exhaustive chemical characterization of each extract, but rather the establishment of a rapid and reliable methodology for screening and prioritization. In this context, phytochemical profiling provides useful information on the major classes of metabolites present in the extracts, which, when combined with bibliographic data (Table 1), may give preliminary indications on potential bioactivities. Although the predominance of a chemical family does not necessarily imply bioactivity, these data offer working hypotheses that may guide future bio‐guided investigations.

Although the bioactivity scores provide a useful comparative measure, they are limited by the lack of independent biological replicates, as well as by extraction efficiency variations across different plant types. For example, the succulent texture of *Portulaca oleracea* or the resinous nature of *Cormus domestica* buds may have hindered full recovery of bioactive compounds using primarily a polar extraction medium. As a result, their bioactivity scores may underestimate their true potential.

Furthermore, beyond the absolute bioactivity score, the novelty of an observed activity plays a critical role in determining a plant's value for cosmetic innovation. Even if a plant extract exhibits moderate or low inhibition in a given assay, the absence of prior literature or market references reporting such activity suggests that the finding is novel. This novelty factor represents a significant opportunity, as an extract demonstrating a previously unreported biological effect, regardless of its initial intensity, can serve as a foundation for further optimization and industrial development.

Therefore, in parallel, an innovation score (0–100) was introduced, quantifying the degree of novelty by comparing the observed activities to existing patent databases, commercial ingredients, and peer‐reviewed literature (see Section 4 for details). For each species, the innovation score was obtained by summing three sub‐scores (market, patent, and literature presence, each 0–3). The individual sub‐scores reflect how frequently the activity has already been reported or commercialized: Low scores (0–1) indicate existing claims, whereas high scores (2–3) suggest novelty. The total score was then normalized to a 0–100 scale. Table 3 reports these scores across all tested species.

**TABLE 3:** Summary of the innovative score of each plant species.

Botanical name	Market	Patent	Literature	Score
*Achillea millefolium*	0	1	1	22
*Agrimonia eupatoria*	0	0	2	22
*Baquifoliumaquifolium*	1	3	1	56
*Betula pendula*	1	1	2	44
*Borago officinalis*	0	1	0	11
*Capsella bursa‐pastoris*	3	3	1	78
*Clematis vitalba*	2	2	2	67
*Cormus domestica*	3	2	2	78
*Corylus avellana*	2	2	2	67
*Dipsacus fullonum*	2	1	2	56
*Lactuca serriola*	0	1	3	44
*Narcissus jonquilla*	3	2	1	67
*Plantago lanceolata*	0	0	1	11
*Plantago major*	0	1	0	11
*Polygonatum multiflorum*	1	1	2	44
*Portulaca oleracea*	0	0	1	11
*Primula veris*	2	2	2	67
*Spartium junceum*	3	3	1	78

This scoring system relies on species already included in Chinese cosmetic ingredient lists, which may introduce a bias toward relatively well‐documented plants. Less‐studied species not included in these lists could possess unrecognized innovative potential that is not captured here.

This two‐tiered system allows identification of extracts that are not only biologically active but also underrepresented in existing scientific and commercial ingredients. Importantly, some species may display a mismatch between the two scores, which can be informative. For instance, *Narcissus jonquilla* reached one of the highest innovation scores (78/100), suggesting that the activities observed are largely absent from scientific or commercial records. Yet, its bioactivity score was notably low (12/100), reflecting weak inhibition across all assays. This discrepancy illustrates that a high innovation score, on its own, does not guarantee interest. In the case of *N. jonquilla*, the low activity may come from the presence of alkaloids such as lycoramine, which are known for their toxicity when ingested [15, 40]. Although such compounds may be used at controlled concentrations in cosmetics, particularly in perfumery, their known toxicity may limit broader applications. This example underscores the importance of combining innovation potential with considerations of bioactivity and safety.

**FIGURE 3:**
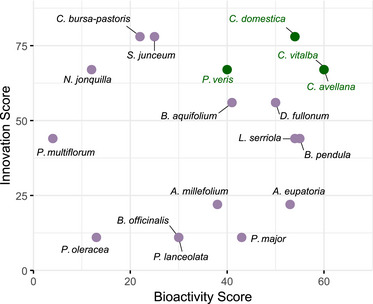
Scatter plot of bioactivity (*X*‐axis) and innovation scores (*Y*‐axis) for the different plant species.

To better visualize the balance between bioactivity and novelty, Figure 3 presents a two‐dimensional scatter plot of innovation (*Y*‐axis) versus bioactivity (*X*‐axis) scores. Each point represents one plant species, positioned according to its score in both dimensions.

The *X*‐axis (bioactivity score) reflects the normalized performance of each extract across all in vitro assays (see Section 4), on a scale from 0 to 100, where higher values indicate stronger inhibition or antioxidant effects. The *Y*‐axis (innovation score) represents the degree of novelty, also scaled from 0 to 100, as described in the previous section.

This visualization enables a rapid identification of candidates that are both effective and underexploited. Extracts located in the upper right quadrant combine strong bioactivity and high innovation potential and are therefore prioritized. Although the bioactivity score may be improved through formulation and extraction optimization, the innovation score offers an early indicator of unexplored value.

On the basis of this rationale, plants with innovation scores ≥60/100 and bioactivity scores ≥40/100 were considered the most promising. Although the threshold values are arbitrary, they helped narrow down a short list of high‐potential species.

The most promising candidate plants identified through this dual‐scoring approach include the following:

- the leaves of old‐man's‐beard (*C. vitalba*)
- the buds of the service tree (*C. domestica*)
- the leaves of common hazel (*C. avellana*)
- the aerial parts of cowslip primrose (*P. veris*)

These species are not currently widely used in cosmetics, and the literature describing their cosmetic‐relevant bioactivities remains relatively limited, suggesting untapped potential. However, further steps are needed to validate and optimize their use. This includes refining extraction procedures, selecting appropriate formulation systems, and confirming efficacy through more advanced biological models. These aspects remain essential to the successful transformation of raw extracts into standardized, efficacious cosmetic ingredients.

Beyond these missing essential translational aspects, another important limitation of the current study concerns cultivation. Plant sourcing parameters, such as geographical origin, harvest timing, and cultivation methods, are known to impact bioactive compound content and, consequently, biological activity [41, 42]. Advances in controlled cultivation techniques over the past few years offer new opportunities to enhance metabolite production and/or activity and ensure standardization, yet these aspects were not included in the dual scoring system.

To explore this further, the following section examines how cultivation conditions could influence the potential of a wild species, *B. officinalis*, as a cosmetic ingredient. This species was specifically chosen for its moderate bioactivity and innovation scores, which made it a relevant model to test whether cultivation can enhance the value of species initially considered less promising. Additionally, its known adaptability to indoor farming, identified in collaboration with an industrial partner, supported its selection for this case study. Through controlled cultivation using rotational geoponics, its bioactive potential was reassessed, providing a concrete example of how cultivation strategies can revalorize otherwise overlooked or underused species, complement early‐stage screening approaches, and open up new development pathways.

### 2.3 Indoor Cultivation of *B. officinalis*: Feasibility and Potential for Cosmetic Applications

During the selection and screening process, certain plant candidates were excluded due to factors such as low observed biological activity or the presence of already well‐documented properties. Although extraction optimization is a well‐established strategy for enhancing the bioactive content of plant extracts [43, 44, 45, 46], an additional key factor influencing metabolite production is the cultivation method. Advances in cultivation strategies, particularly those leveraging green biotechnologies, have demonstrated a significant impact on the biosynthesis of bioactive compounds [47]. By refining cultivation conditions, it is possible to modulate the plant's metabolic pathways, potentially increasing the production of secondary metabolites associated with biological activity [48, 49, 50]. Thus, considering cultivation strategies at earlier stages may bring a new dimension to the process of plant selection for cosmetic applications.

**FIGURE 4:**
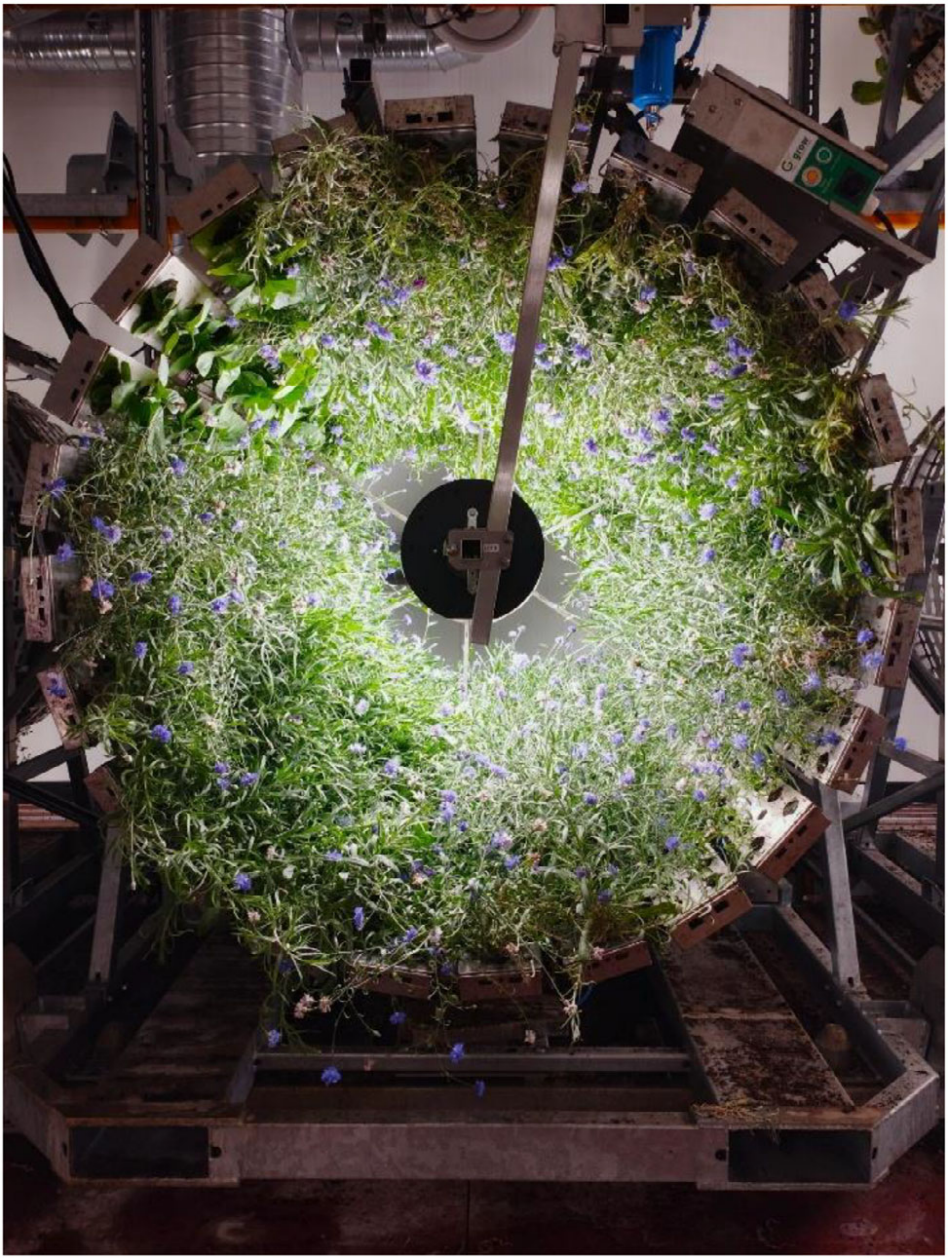
Example of a rotational cultivation system.

Among these emerging approaches, rotational cultivation presents a promising technique for controlled‐environment indoor cultivation. This system involves a rotating structure that improves plant exposure to light, nutrients, and water (Figure 4). Unlike many vertical farming systems that rely solely on hydroponics or aeroponics, this technology incorporates a substrate with soil, providing a growth medium that more closely mimics natural conditions. The presence of soil distinguishes this approach by supporting more complex root–microbe interactions and nutrient dynamics, which may contribute to a richer secondary metabolite profile.

By precisely regulating key growth parameters, such as temperature, humidity, light intensity, and photoperiod, rotational cultivation can improve resource efficiency, reducing both land use and water consumption compared to conventional agriculture [51]. Moreover, by modulating parameters such as temperature, photoperiod, and nutrient availability and introducing stimuli like water stress, it is possible to influence the plant's metabolic processes, thereby modifying (increasing or decreasing) the production of secondary metabolites, which are often directly linked to the observed biological activity. This technology offers a promising opportunity to enhance the properties of various plant species, particularly those that exhibit limited bioactive compound production in the wild and may otherwise have been overlooked for cosmetic applications.

*B. officinalis*, a fifth species not initially prioritized but selected for its moderate bioactivity and innovation scores and high feasibility for indoor cultivation, is an annual herb of the Boraginaceae family. It was used as a species to explore the impact of rotary geoponics on bioactive compound production and extraction efficiency. Widely cultivated for its medicinal and culinary applications [52], borage is native to the Western Mediterranean but has since been introduced to various regions, including Eastern Europe and the Americas [53]. In France, it is found throughout most of the territory, making it a relevant species for studying the potential of native flora in cosmetic applications. Moreover, its prior validation during the screening process confirmed its compatibility with current cosmetic regulations and biodiversity conservation frameworks [5, 7, 10, 11].

The biological activity assays conducted during the screening phase of this work revealed that hydroalcoholic extracts from borage aerial parts harvested in the wild exhibited moderate antioxidant (40.3% ± 0.1% activity in the DPPH free radical scavenging assay), anti‐hyaluronidase (48.8% ± 5.3% inhibition), and anti‐lipoxygenase (36.7% ± 0.1% inhibition) activities. These bioactivities, however, have already been well documented in the scientific literature and are incorporated into existing cosmetic ingredients [54, 55, 56, 57]. A market analysis further confirmed that borage‐based active ingredients, primarily derived from its aerial parts, are widely available and valued for their antioxidant, anti‐inflammatory, and moisturizing properties.

Given the growing interest in controlled‐environment agriculture as a means of optimizing the bioactive potential of plants, this work investigated whether rotational cultivation in a controlled setting could enhance *B. officinalis* in three key areas: (i) biological activity, (ii) biomass production yield, and (iii) extraction yield from extracted biomass. By precisely adjusting cultivation conditions, this method may influence the plant's metabolic pathways, potentially leading to increased secondary metabolite production. The objective was to assess whether this approach could yield a more potent bioactive ingredient or improve extraction efficiency, offering an alternative strategy for the sustainable and optimized use of borage in cosmetics.

In this study, *B. officinalis* was cultivated under controlled conditions using a rotary geoponic system. Separate culture trays were subjected to environmental variations to assess their impact on the biological activity of ethanolic extracts from the aerial parts harvested before flowering. Specifically, a subset of plants was grown under reduced temperature conditions—6°C lower both during the day and at night—whereas control plants were maintained under standard temperature conditions (24°C during the day and 21°C at night). Additional groups cultivated under low temperature were also exposed to modified irrigation conditions to simulate water stress.

**FIGURE 5:**
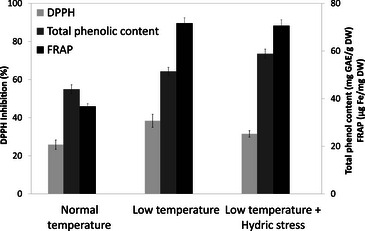
Antioxidant capacity of *Borago officinalis* extracts under different cultivation conditions. Each condition was tested in triplicate (*N* = 3, technical replicates); no independent biological replicates were performed; results are indicative; data are expressed as mean ± SEM. FRAP, ferric‐reducing antioxidant power.

Ethanolic extracts obtained from each cultivation condition were analyzed for antioxidant properties, including ferric‐reducing antioxidant power (FRAP), total phenolic content (TPC), and DPPH radical scavenging activity (Figure 5). Results are expressed as the mean ± standard error of the mean (SEM) of three technical replicates (*N* = 3), with each extract tested in triplicate wells. Cultivation under reduced temperature led to a 95% increase in FRAP values (71.7 ± 2.2 µg Fe/mg DW) compared to the control (36.7 ± 1.2 µg Fe/mg DW). Similarly, TPC rose by 17% (51.4 ± 1.7 mg GAE/g DW vs. 43.9 ± 1.9 mg GAE/g DW). The DPPH assay also showed enhanced radical scavenging activity, with inhibition percentages increasing from 25.8% ± 2.4% (control) to 38.4% ± 3.4% under low temperature. It should be noted that these results are based on technical replicates only; no independent biological replicates were performed. Therefore, although the trends observed are indicative, further studies using independent plant collections would be needed to confirm the statistical robustness of these findings.

These preliminary findings indicate that temperature modulation during cultivation may positively influence the antioxidant potential of *B. officinalis*, possibly by stimulating the biosynthesis of secondary metabolites.

Interestingly, when water stress was introduced alongside low temperature, no further improvement in antioxidant capacity was observed: TPC reached 58.9 ± 1.9 mg GAE/g DW, FRAP values were 70.6 ± 2.5 µg Fe/mg DW, and DPPH inhibition was 31.5% ± 1.7%. This suggests that under cooler conditions, *B. officinalis* displays a degree of resilience to additional hydric stress, with no cumulative benefit on antioxidant performance.

**FIGURE 6:**
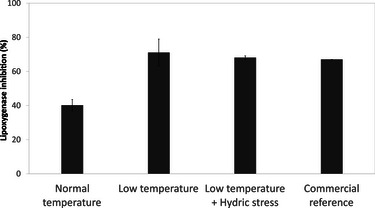
Lipoxygenase inhibition (%) of *Borago officinalis* extracts cultivated under different conditions, compared to a commercial benchmark ingredient marketed for anti‐inflammatory activity (non‐borage plant extract). Each condition was tested in triplicate (*N* = 3, technical replicates); no independent biological replicates were performed; results are indicative; data are expressed as mean ± SEM.

These findings reinforce the observation that temperature modulation can serve as a strategic lever for enhancing plant bioactivity, particularly antioxidant potential. To further investigate this effect, the impact of cultivation temperature on anti‐inflammatory activity was evaluated through lipoxygenase inhibition assays (Figure 6). Extracts obtained from plants cultivated under reduced temperature conditions exhibited significantly higher inhibitory activity (71.1% ± 7.9% inhibition) compared to the control condition (40.2% ± 3.3%). This level of activity was comparable to that of a commercial cosmetic ingredient marketed for its anti‐inflammatory properties, tested under the same conditions, and used here as a benchmark.

As observed in antioxidant assays, the addition of water stress to the reduced temperature regime did not lead to further improvements in activity. The inhibition percentage under combined abiotic stress conditions was 68.1% ± 1.0%, suggesting that *B. officinalis* displays resilience to simultaneous environmental stresses.

Although metabolomic profiling was not performed in this study, previous reports indicate that *B. officinalis* contains several classes of bioactive compounds, notably flavonoids, phenolic acids, sterols, and fatty acids such as γ‐linolenic acid. The antioxidant and anti‐inflammatory activities observed here are most likely linked to the phenolic constituents, such as rosmarinic and syringic acid, as reported in the literature [25]. Importantly, abiotic stress such as reduced growth temperature is known to stimulate secondary metabolism, in particular the phenylpropanoid pathway, thereby enhancing the accumulation of phenolic metabolites [58]. This provides a plausible explanation for the enhanced bioactivities observed in *B. officinalis* cultivated under temperature‐modulated conditions and suggests directions for future metabolomic analyses.

Overall, these results underscore the potential of temperature as a key environmental parameter for modulating the bioactivity of *B. officinalis*. However, from an industrial standpoint, the feasibility of applying extreme cultivation conditions must be carefully considered, as substantial cooling (or heating) could lead to increased energy costs. A recent study on rotational cultivation of lettuce using the same system as the one employed here (Futura Gaïa) highlighted that energy consumption remains a major challenge for indoor farming, with cooling and ventilation already accounting for a significant proportion of electricity demand of approximately 37%, even under moderate temperature conditions (24°C during the day/19°C at night) [51]. A further reduction in growing temperatures, as in our case (18°C during the day/15°C at night), could increase this energy load, although precise quantification remains to be established. These results highlight that any gains in bioactivity achieved through temperature modulation must be balanced against operational constraints. Future work should therefore incorporate technical‐economic and life‐cycle assessments to identify cultivation strategies that maximize bioactivity while maintaining sustainability.

Additional environmental variables such as light intensity, photoperiod, and nutrient availability may also influence bioactivity and could benefit from further investigation. Advanced experimental frameworks, including response surface methodology and AI‐assisted modeling, could support the identification of ideal cultivation conditions. Applying this approach to other medicinal and cosmetic plants may broaden the development of sustainably produced, bioactive plant‐based ingredients.

The case study of *B. officinalis* illustrates how cultivation strategies can substantially influence biological potential, thereby expanding the scope of the dual‐scoring methodology. Nevertheless, moving from promising extracts to functional cosmetic ingredients requires addressing further key steps. Extraction protocols must be optimized to maximize activity, appropriate formulation systems must be developed to guarantee stability and efficacy, and validation in advanced biological models is essential to confirm relevance. In addition, industrial translation requires consideration of safety evaluation. Together, these aspects highlight that although screening and cultivation represent crucial entry points, a broader translational framework is necessary to unlock the full potential of novel botanical resources for cosmetic applications.

## 3 Conclusions

This work aimed to identify innovative cosmetic active ingredients derived from plants present in France through a systematic screening strategy. A multi‐criteria preselection process was first applied to narrow down candidates from the 1614 plants authorized in Chinese cosmetic regulations. Eighteen species were retained for further evaluation based on their regulatory compatibility and cosmetic potential within a global development framework.

The selection process combined untargeted phytochemical profiling using HPLC–DAD/ELSD with in vitro chemical and enzymatic assays to assess biological activity. Innovation potential was also considered, integrating factors such as market presence, existing patents, and scientific literature coverage. Special attention was given to sustainability and regulatory constraints to ensure candidate viability from both efficacy and ethical standpoints.

From this screening, four standout species emerged: the leaves of old‐man's‐beard (*C. vitalba*), the buds of the service tree (*C. domestica*), the leaves of common hazel (*C. avellana*), and the aerial parts of cowslip primrose (*P. veris*). These plants are widely present in France and demonstrate promising biological activities, positioning them as valuable resources for developing effective, natural cosmetic ingredients. Future research should aim to validate their biological activities in more complex systems and identify their bioactive constituents, particularly through bio‐guided fractionation, to support extract optimization and claim substantiation.

Although the screening identified promising candidates, this study also highlights the potential for innovation through the use of modern cultivation methods to enhance plant quality and bioactivity. A complementary investigation focused on *B. officinalis*, a species that initially demonstrated limited bioactivity but, when cultivated under controlled conditions using rotational cultivation at reduced temperatures, showed significant improvements in its antioxidant and anti‐inflammatory potential. These findings highlight the potential of innovative agricultural practices to enhance the metabolic expression and cosmetic relevance of well‐known or underexploited plant species, potentially expanding the range of viable candidates for cosmetic development.

Importantly, this work primarily aimed to establish a methodological framework for plant‐based cosmetic screening, rather than to provide a definitive or exhaustive list of innovative species. The methodology is flexible and can be adapted to different regulatory contexts or innovation priorities. For example, although the current study focused on species already included in Chinese cosmetic ingredient lists, ensuring regulatory feasibility for a worldwide development, future applications of this workflow could modify the selection criteria to exclude this regulatory framework, therefore including less‐documented or underexplored plants. Such modifications would allow identification of highly novel species that may have been overlooked, thereby mitigating the potential bias of the criteria here chosen and broadening the scope of innovation.

In addition, although chromatographic profiling by HPLC–DAD/ELSD proved useful for rapid and comparative screening, it does not enable the identification of individual bioactive metabolites. Future applications could therefore benefit from the integration of complementary techniques such as LC–(HR)MS and bio‐guided fractionation, which would provide a more precise characterization of active compounds and further strengthen the link between phytochemical fingerprints and biological effects.

Together, these findings advocate for an integrated approach that combines effective plant screening with advanced cultivation strategies to support the development of safe, sustainable, and high‐performance plant‐based cosmetic ingredients.

## 4 Experimental Section

### 4.1 Reagents

Ultra‐pure water was obtained from distilled water using an Elix Advantage 3 purification system (Merck Millipore, Darmstadt, Germany). Ethanol (96%) was purchased from VWR Chemicals. Methanol and formic acid (HPLC grade) were supplied by Sigma‐Aldrich (Saint Louis, USA). All reagents for biological activity assays were purchased from Sigma‐Aldrich unless otherwise stated. FALGPA (2‐furanacryloyl‐l‐leucylglycyl‐l‐prolyl‐l‐alanine) was obtained from BACHEM (Bubendorf, Switzerland).

### 4.2 Plant Materials

Plant resources were collected during spring 2022 and 2023 through a network of collaborators primarily located in South‐Eastern France. Detailed information on plant species, harvested organs, and collection sites is presented in Table 4. Although voucher specimens were not deposited, plant identification was carried out at the time of collection by an experienced botanist (Jean‐Louis Polidori) and confirmed using authoritative taxonomic databases. This represents a limitation in terms of long‐term traceability. In cases involving cultivated plants, identification was performed on the basis of the taxonomic verification of the seed lots used.

**TABLE 4:** Details of plant material collection.

Botanical name	Organs sourced	Location
*Achillea millefolium*	Aerial parts	Isola village
*Agrimonia eupatoria*	Aerial parts	Nice
*Baquifolium aquifolium*	Aerial parts	Lille
*Betula pendula*	Aerial parts	Mouans‐Sartoux
*Borago officinalis*	Aerial parts	Saint‐Laurent‐du‐Var
*Capsella bursa‐pastoris*	Aerial parts	Isola village
*Clematis vitalba*	Leaves	Isola village
*Cormus domestica*	Buds	Mouans‐Sartoux
*Corylus avellana*	Leaves	Mouans‐Sartoux
*Dipsacus fullonum*	Aerial parts and roots	Mouans‐Sartoux
*Lactuca serriola*	Leaves	Isola village
*Narcissus jonquilla*	Aerial parts	Nice
*Plantago lanceolata*	Aerial parts	Nice
*Plantago major*	Aerial parts	Saint Etienne de Tinée
*Polygonatum multiflorum*	Roots/Rhizome	Mérinchal
*Portulaca oleracea*	Aerial parts and roots	Mouans‐Sartoux
*Primula veris*	Aerial parts	Saint Etienne de Tinée
*Spartium junceum*	Flowers	Isola village

Rotational cultivation of *B. officinalis* was carried out using seeds from Voltz. Germination was conducted in soil composed of coconut fiber, peat, and perlite (Florentaise, Lavilledieu, France), and seedlings were later transplanted into trays filled with soil made from wood fiber, bark fiber, and clay (Florentaise, Lavilledieu, France). The trays were mounted on rotary cultivation systems rotating at one revolution per 50 min. Illumination was provided by three lamps delivering 250 µmol/(m^2^ s) for 16 h/day, followed by 8 h of darkness. Plants received a nutrient solution containing macronutrients (N, P, K, Ca, Mg) and micronutrients (Cu, Fe, Mn, Mo, Zn). For water‐stress conditions, irrigation was reduced by 25% during the final 10 days of cultivation. Harvesting occurred 26 days post‐transplantation, prior to flowering. Aerial parts were dried and ground for extraction.

### 4.3 Extraction Procedures

For the screening study, hydroalcoholic extracts were prepared by macerating ground plant material (1:10 w/w plant‐to‐solvent ratio, ethanol/water 1:1 w/w) for 2 h at room temperature under magnetic stirring (500 rpm). The mixtures were filtered (Labbox 200 µm thick, 10–12 µm pore size filter paper), and the solvents evaporated under reduced pressure. For cosmetic‐grade liquid extracts (LEs), 99.5% propylene glycol was used at a 1:10 w/w ratio. Ground plant material was stirred at room temperature for 7 h (500 rpm) and then filtered through 50 µm filter bags to remove solids.

Dried *B. officinalis* biomass from rotary cultivation was extracted using ethanol at a 1:1 w/w plant‐to‐solvent ratio. Extraction was performed under magnetic stirring (500 rpm) for 2 h at room temperature. The mixture was filtered using 200 µm thick filter paper (10–12 µm pore size; Labbox), and the solvent was removed under reduced pressure using a rotary evaporator.

### 4.4 High‐Performance Liquid Chromatography

Samples were prepared at 10 mg/mL in methanol, water, methanol/water, or methanol/isopropanol mixtures and filtered through 0.45 µm polyethersulfone non‐sterile filters (Labbox). All solvents used were HPLC grade (Sigma‐Aldrich). Analyses were carried out using an Agilent 1200 Series HPLC system (Courtaboeuf, France) equipped with a DAD and an ELSD. ELSD settings were 40°C, nitrogen pressure at 4.5 bar, and gain set to 4. Chromatographic separation was achieved on a Phenomenex Luna C18(2) column (250 × 4.6 mm^2^, 5 µm, 100 Å), with a guard column. Injection volume was 10 µL, and the flow rate was 1 mL/min. The mobile phase consisted of water with 0.1% formic acid (A), methanol (B), and isopropanol (C). The elution gradient was as follows: 0–15 min: 5%–50% B, 15–20 min: 50% B, 20–30 min: 75%, 30 min: 75% B, 35–40 min: 75%–100% B, 40–45 min: 100% B, 45–50 min: 0%–100% C, 50–55 min: 100% C. System operation and data processing were managed using ChemStation software (Agilent).

### 4.5 Assessment of Biological Activities

DEs and commercial references were dissolved in DMSO at 3.433 mg DE/mL, corresponding to a final concentration of 100 µg/mL per well in 96‐well plates. LEs were diluted to the same concentration (3.433 mg LE/mL) in DMSO. For the anti‐lipoxygenase assay, non‐sterile flat‐bottom clear acrylic 96‐well plates (Corning UV‐transparent, Sigma‐Aldrich) were used. For all other enzymatic and chemical assays, non‐sterile flat‐bottom polystyrene 96‐well plates (Greiner, Sigma‐Aldrich) were employed. During incubation or shaking steps, plates were sealed using transparent non‐sterile EASYSeal sealing films (Greiner). Pipetting was performed with an Eppendorf epMotion 5073 liquid handler using epT.I.P.S. Motion pipette tips (0.5–50 and 5–300 µL). Optical density (OD) readings were obtained using a SpectraMax Plus 384 spectrophotometer (Molecular Devices, UK) in plate format, and data were processed using SoftMax Pro software (Molecular Devices, UK). Each extract was tested in three technical replicates (pipetted into three independent wells), and results are expressed as mean ± SEM, with *N* = 3. Descriptive statistics were calculated using Prism (GraphPad Software, USA).

For the anti‐hyaluronidase and anti‐collagenase assays, inhibition percentages were calculated using the following formula:

$$\%\;I=\frac{OD_{\mathrm{sample}}}{OD_{\mathrm{blank}}-OD_{\mathrm{control}}}\times 100$$

For all other assays, inhibition percentages were calculated as follows:

$$\%\;I=\frac{OD_{\mathrm{control}}-OD_{\mathrm{sample}}}{OD_{\mathrm{control}}}\times 100$$

For all assays, OD values were corrected by subtracting the final OD from the initial OD:

$$OD_{\mathrm{sample}}\mathrm{or}\;OD_{\mathrm{control}}\mathrm{or}\;OD_{\mathrm{blank}}=\;OD_{\mathrm{first}\;\mathrm{reading}}-\;OD_{\mathrm{second}\;\mathrm{reading}}$$

All data represent the mean ± standard deviation (SD) of three independent experiments. Protocols were based on previously published methods with minor modifications [59, 60].

### 4.6 Brightening Activity

Tyrosinase inhibition was evaluated by adding 150 µL of mushroom tyrosinase (171.66 U/mL in phosphate buffer pH 6.8) to each well (final enzyme concentration: 100 U/mL), followed by 7.5 µL of test extracts (final concentration: 100 µg/mL). DMSO served as the negative control. After 2 min of agitation and 20 min of incubation in the dark at room temperature, the first OD was recorded at 480 nm. Subsequently, 100 µL of l‐tyrosine (1 mM in phosphate buffer, pH 6.8; final concentration: 0.388 mM) was added, followed by 2 min agitation and 20 min incubation in the dark. A second OD reading was taken at 480 nm.

### 4.7 Anti‐Inflammatory Activity

Lipoxygenase inhibition was assessed by adding 150 µL of enzyme solution (686.66 U/mL in phosphate buffer pH 8; final concentration: 400 U/mL) to each well, followed by 7.5 µL of test extract (100 µg/mL final concentration). After agitation and 10 min incubation in the dark at room temperature, 100 µL of linoleic acid substrate solution (prepared from 5 µL linoleic acid, 10 µL ethanol, and 20 mL phosphate buffer; final concentration: 0.388 mM) was added. OD was measured at 235 nm after 2 min of agitation and again after 50 min of incubation.

### 4.8 Anti‐Hyaluronidase Activity

Each well received 150 µL of bovine hyaluronidase (13.3 U/mL in pH 7 buffer) and 7.5 µL of extract (final concentration: 100 µg/mL). DMSO and buffer alone were used as negative (OD_control_) and blank (OD_blank_) controls, respectively. After 2 min of agitation and 20 min of incubation at 37°C, 100 µL of hyaluronic acid (150 µg/mL in pH 5.35 buffer) was added. The plate was agitated and read at 600 nm. After 30 min incubation at 37°C, 50 µL of 40 mM CTAB in 2% NaOH was added, followed by 2 min agitation and a second OD reading at 600 nm.

### 4.9 Anti‐Collagenase Activity

Collagenase inhibition was evaluated by adding 150 µL of collagenase solution (53 U/mL in pH 7.5 tricine buffer; final concentration: 31 U/mL) and 7.5 µL of extract (100 µg/mL). DMSO and tricine buffer were used as negative and blank controls, respectively. After 15 min incubation at room temperature, OD was measured at 345 nm. Then, 100 µL of FALGPA (5.15 mM in tricine buffer) was added. Following 2 min of agitation and 30 min incubation, a second OD reading was recorded at 345 nm.

### 4.10 DPPH Free Radical Scavenging Activity

Each well received 150 µL of a 1:1 (v/v) mixture of 0.1 M acetate buffer (pH 5.4) and ethanol, along with 7.5 µL of extract (final concentration: 100 µg/mL). After vortexing in the dark for 2 min, the OD was read at 517 nm. Then, 100 µL of DPPH solution (386.25 µM in ethanol; final concentration: 150 µM) was added. The plate was sealed, vortexed, and incubated in the dark for 30 min before the second OD reading at 517 nm.

### 4.11 Ferric Ion Reducing Antioxidant Potential (FRAP)

The FRAP reagent was freshly prepared by combining 25 mL acetate buffer (0.3 M, pH 3.6), 2.5 mL 2,4,6‐tris(2‐pyridyl)‐*s*‐triazine (TPTZ) solution (10 mM in 40 mM HCl), and 2.5 mL of 20 mM FeCl_3_. Each well received 150 µL of acetate buffer and 7.5 µL of extract (100 µg/mL). FeSO_4_·7H_2_O (1 mg/mL) and DMSO served as positive and negative controls. After vortexing, a first OD reading was taken at 593 nm. Then, 100 µL of FRAP reagent was added. After 4 min of vortexing in the dark, a second OD reading was performed. Antioxidant activities were expressed as µg Fe^2+^ equivalents per mg DE (µg Fe^2+^eq./mg DW), based on a calibration curve from 0.09 to 1.2 mM FeSO_4_·7H_2_O.

### 4.12 Total Phenolic Content

The TPC was determined using the Folin–Ciocalteu method. Each well received 75 µL of ultrapure water and 15 µL of extract (100 µg/mL). Gallic acid and DMSO served as positive and negative controls, respectively. After an initial OD reading at 765 nm, 25 µL of Folin–Ciocalteu reagent diluted 1:1 with ultrapure water was added. Plates were vortexed and left for 6 min in the dark, followed by the addition of 100 µL of 7.5 g/L sodium carbonate solution. Plates were sealed, vortexed, and incubated in the dark at room temperature for 90 min. A second OD reading at 765 nm was then performed. Results were expressed as mg gallic acid equivalents per g DE (mg GAE/g DW), using a calibration curve from 12.5 to 1000 µg/mL gallic acid.

### 4.13 Data Integration and Scoring System

To enable cross‐comparison between species and guide prioritization of promising extracts, two complementary scores were calculated: a bioactivity score and an innovation score. The former integrates biological assay outcomes across multiple extracts and activities, whereas the latter assesses the novelty of observed bioactivities based on market, patent, and scientific literature coverage. These scores provided a semi‐quantitative framework for evaluating the potential of each species for cosmetic development.

### 4.14 Bioactivity Scoring

To summarize bioactivity across the different assays, a simplified “+” symbol classification system was used to semi‐quantitatively express inhibition or antioxidant effects. The number of “+” symbols attributed to each result was based on the percentage of inhibition or activity, and each symbol contributed one point to the raw bioactivity score of the extract. This raw score was normalized by dividing it by the maximum achievable score for the number of assays performed (e.g., 20 for five assays, 16 for four assays), then scaled to a 0–100 index. This standardized bioactivity index was calculated for each extract (hydroalcoholic and propylene glycol), and the mean of both extracts was used to represent the final bioactivity score for each species.

### 4.15 Innovation Scoring

An innovation score was calculated to assess the novelty of observed bioactivities. Three criteria were considered: market presence, patent coverage, and scientific literature documentation. Each criterion was scored from 0 to 3 based on the availability of cosmetic ingredients, patents, or publications claiming similar activities for the same plant or organ. The scoring scales are detailed below:

Market presence: To determine whether the observed enzymatic and chemical activities are already claimed in commercial products, cosmetic ingredient databases such as UL Prospector and SpecialChem were analyzed.

- A score of 0 was assigned if the activity was explicitly attributed to the same plant organ in an existing ingredient, indicating alignment with market offerings but lacking novelty.
- A score of 1 was given if the activity was claimed in a multi‐plant extract without direct attribution to the species in question, if it is associated with a different plant organ, or if it is used in a non‐skincare application.
- A score of 2 was assigned if at least one of the detected activities had not yet been claimed in any ingredient derived from the plant.
- A score of 3 was given if no cosmetic ingredient derived from the plant was found on the market.

Patent coverage: A preliminary review of patent databases was conducted to assess whether similar bioactivities had already been patented.

- A score of 0 was assigned if the activity was already protected under an existing patent.
- A score of 1 was given if patents existed but involved a different plant organ, extract type, or multi‐plant formulation. This score was also applied if the activity was patented for applications outside skincare, such as pharmaceuticals or haircare.
- A score of 2 was assigned if no patents explicitly claimed the bioactivity of interest.
- A score of 3 was given if no relevant patents were identified at all, indicating a potentially unexplored application.

Scientific literature documentation: This criterion assessed the extent to which the identified bioactivity had been reported in peer‐reviewed scientific publications.

- A score of 0 was assigned if the activity had already been documented, along with the identification of the responsible bioactive compound(s).
- A score of 1 was given if the activity was reported, but without specifying the active compound(s).
- A score of 2 was assigned if no prior studies had identified that specific activity in the plant, but scientific studies on the species existed (e.g., other bioactivities had been reported, or the plant had been analyzed for different purposes).
- A score of 3 was given if no scientific publications evaluating the plant's activity were found, indicating a significant gap in research.

The total score (max. 9) was normalized to a 0–100 scale. This innovation score reflects the degree to which a species may offer unexplored potential for new cosmetic actives.

## Author Contributions

**Maria Viéytez**: conceptualization and methodology, investigation, data curation and formal analysis, writing – original draft. **Aline Robert‐Hazotte**: conceptualization and methodology, proofreading, review, editing, supervision. **Amélie Thomas**: investigation, proofreading, review, editing. **Véronique Nardello‐Rataj**: conceptualization and methodology, proofreading, review, editing, supervision. **Xavier Fernandez**: conceptualization and methodology, proofreading, review, editing, supervision. All authors have read and agreed to the published version of the manuscript.

## Conflicts of Interest

The authors declare no conflicts of interest.

## Supporting information




**Supporting File 1**: cbdv70581‐sup‐0001‐SuppMat.docx

## Data Availability

The data that support the findings of this study are available from the corresponding author upon reasonable request.
