# Supplementary material for: A Workflow for Selecting, Profiling, and Optimizing Plant Extracts for Cosmetic Applications
Source: Chem Biodivers. 2025 Oct 22;22(12):e02397. doi: 10.1002/cbdv.202502397 (PMC12716007; doi:10.1002/cbdv.202502397)
Supplement: Supplementary file 1 — Supporting File 1: cbdv70581‐sup‐0001‐SuppMat.docx [file CBDV-22-e02397-s001.docx]

A Workflow for Selecting, Profiling, and Optimizing Plant Extracts for Cosmetic Applications

SUPPORTING INFORMATION

Maria Viéytez,^a^ Aline Robert-Hazotte,^b^ Amélie Thomas,^c^ Véronique Nardello-Rataj^d^,
and Xavier Fernandez*^,a^

^a^ Université Côte d’Azur, CNRS, UMR 7272, Institut de Chimie de Nice, Parc Valrose, 06108 Nice Cedex 2, France, xavier.fernandez@univ-cotedazur.fr

^b^ Shiseido Europe Innovation Center, 4 Avenue du Général de Gaulle, 45140 Ormes, France

^c^ Futura Gaia Technologies, Chemin du Pont Des Iles, Mas De Polvelière, 30230 Rodilhan, France

^d^ Centrale Lille, Université de Lille, Université Artois, CNRS, UMR 8181, Unité de Catalyse et Chimie du Solide, Lille, France

**HPLC-DAD/ELSD chromatograms of the 18 plants screened**


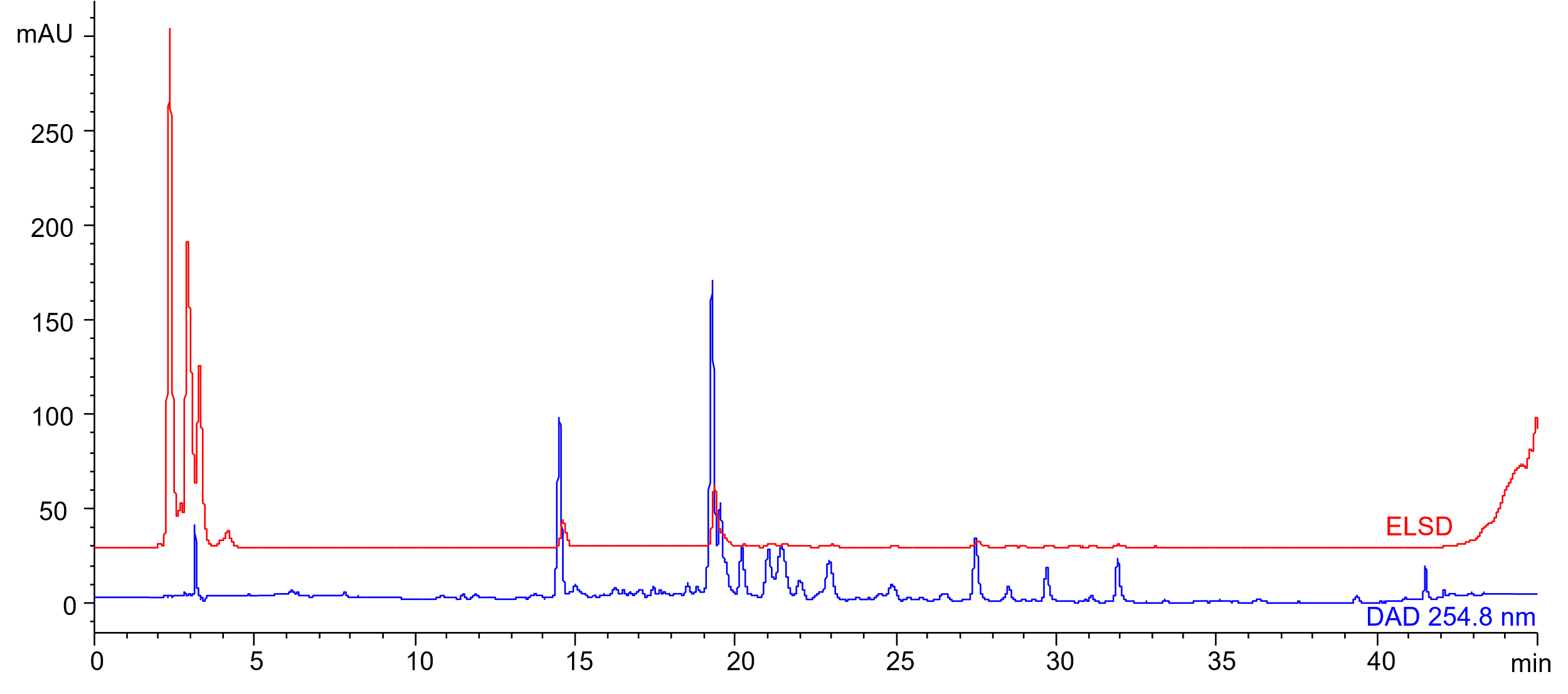


**Figure 1**. HPLC-DAD/ELSD chromatograms of the hydroalcoholic extract of *Achillea millefolium* L.


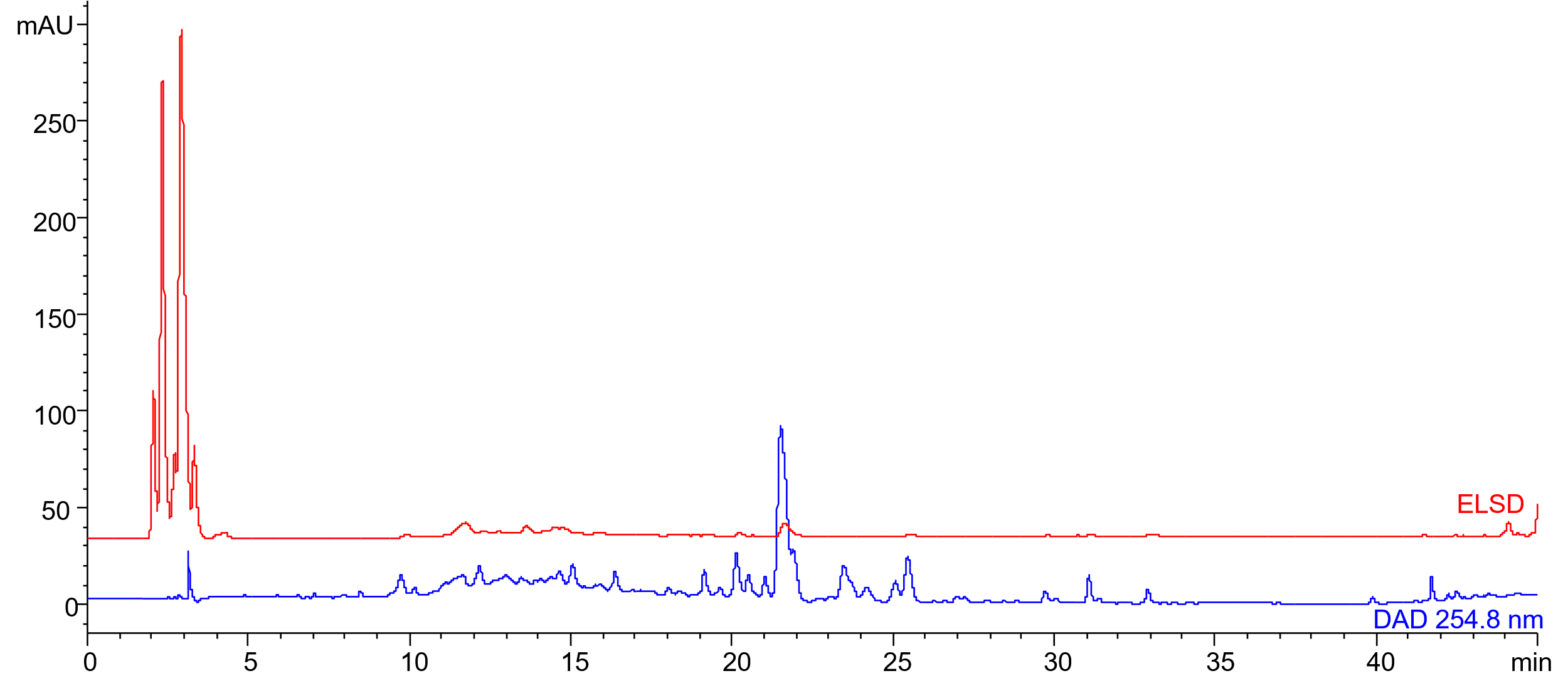


**Figure 2**. HPLC-DAD/ELSD chromatograms of the hydroalcoholic extract of *Agrimonia eupatoria* L.


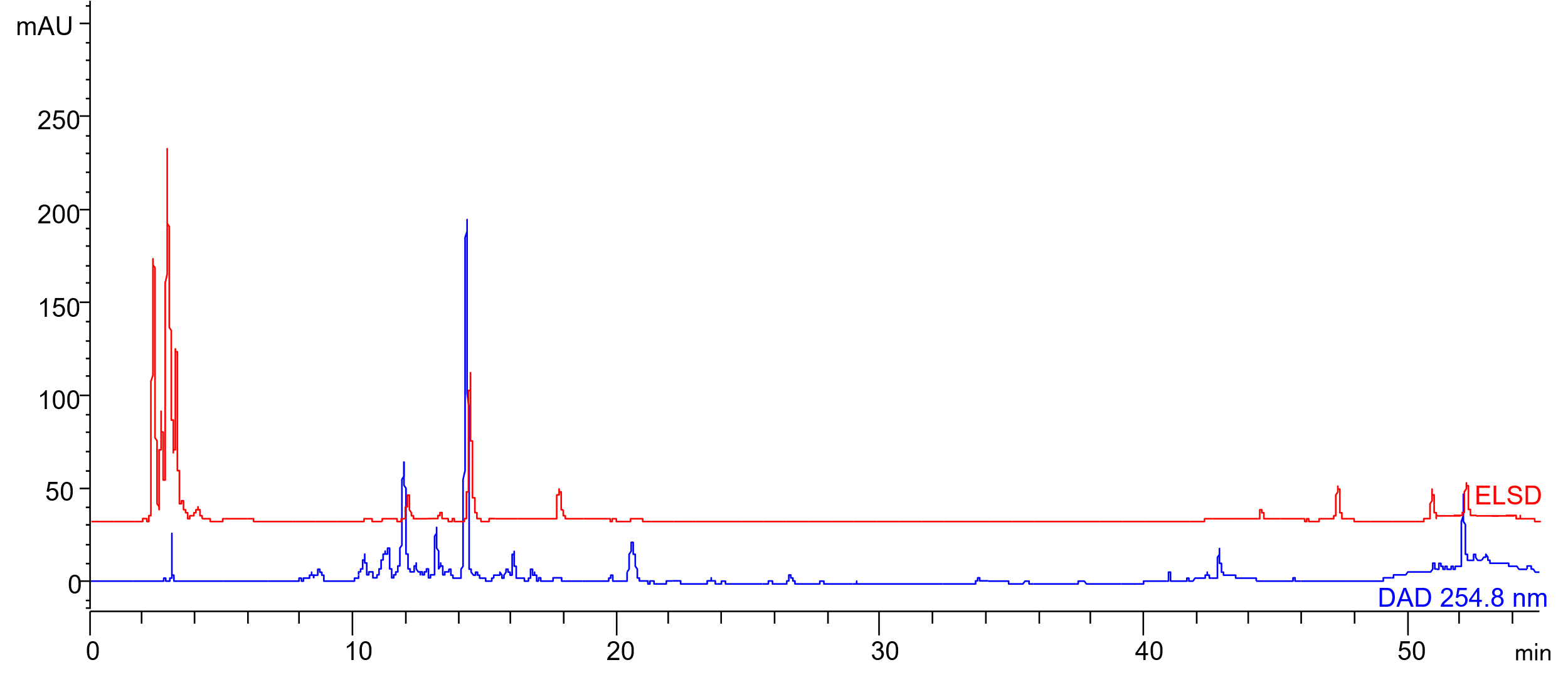


**Figure 3**. HPLC-DAD/ELSD chromatograms of the hydroalcoholic extract of *Berberis aquifolium* L.


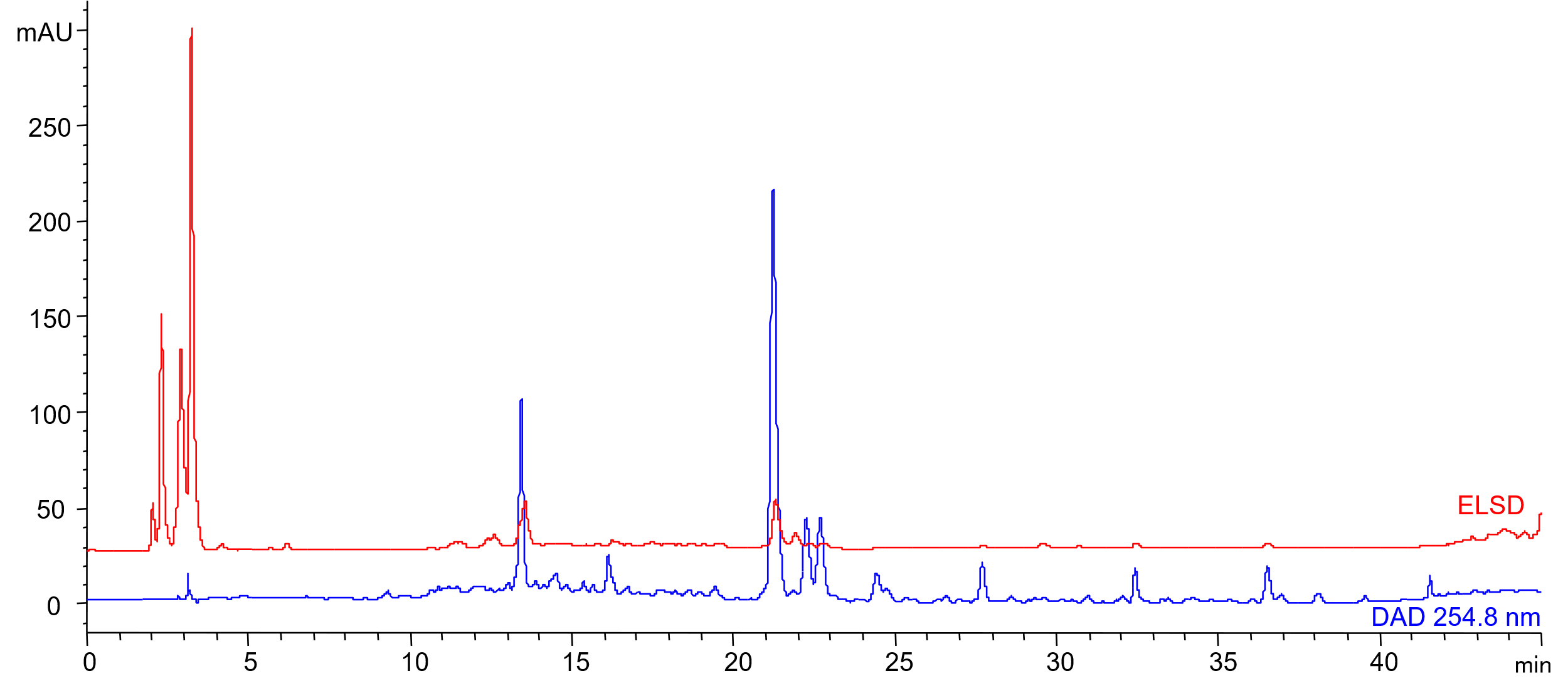


**Figure 4**. HPLC-DAD/ELSD chromatograms of the hydroalcoholic extract of *Betula pendula* Roth


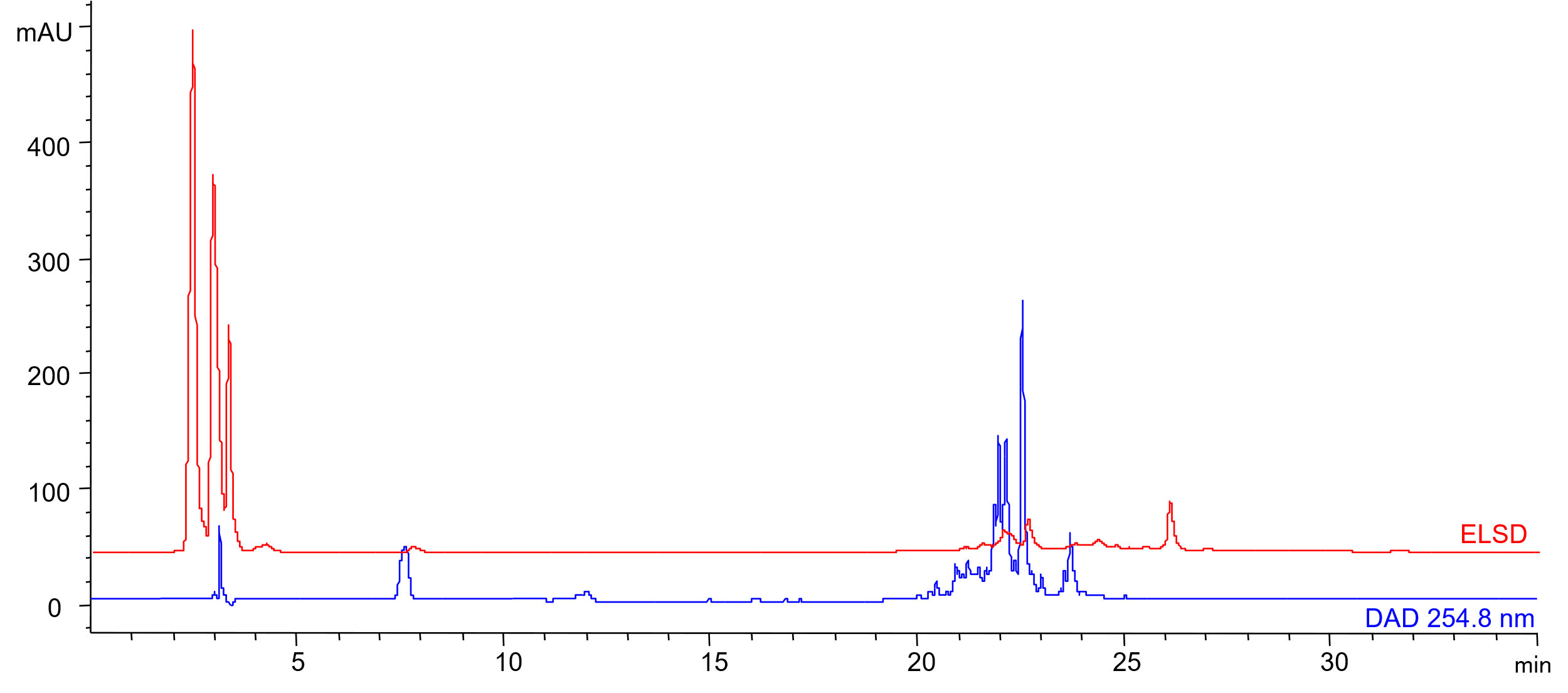


**Figure 5**. HPLC-DAD/ELSD chromatograms of the hydroalcoholic extract of *Borago officinalis* L.


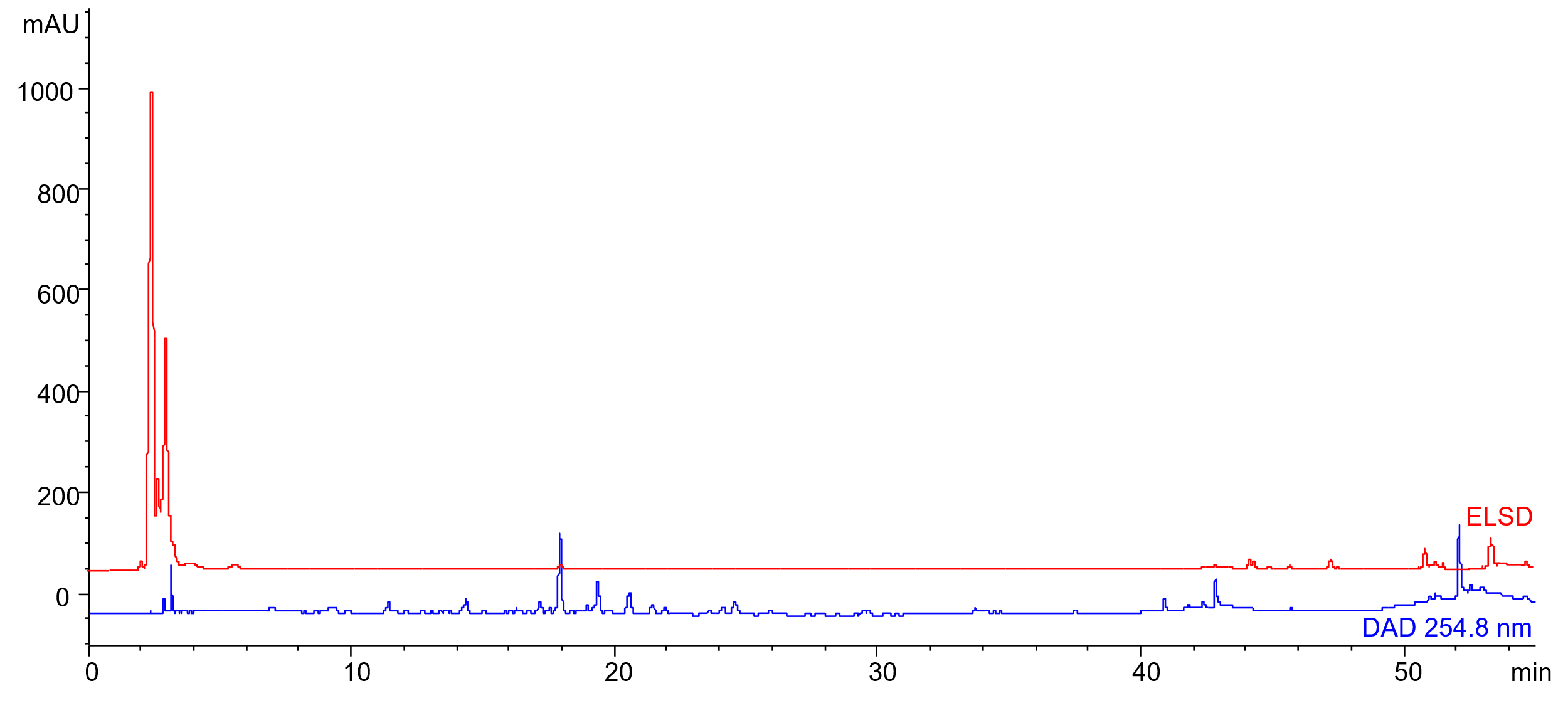


**Figure 6**. HPLC-DAD/ELSD chromatograms of the hydroalcoholic extract of *Capsella bursa-pastoris* (L.) Medik


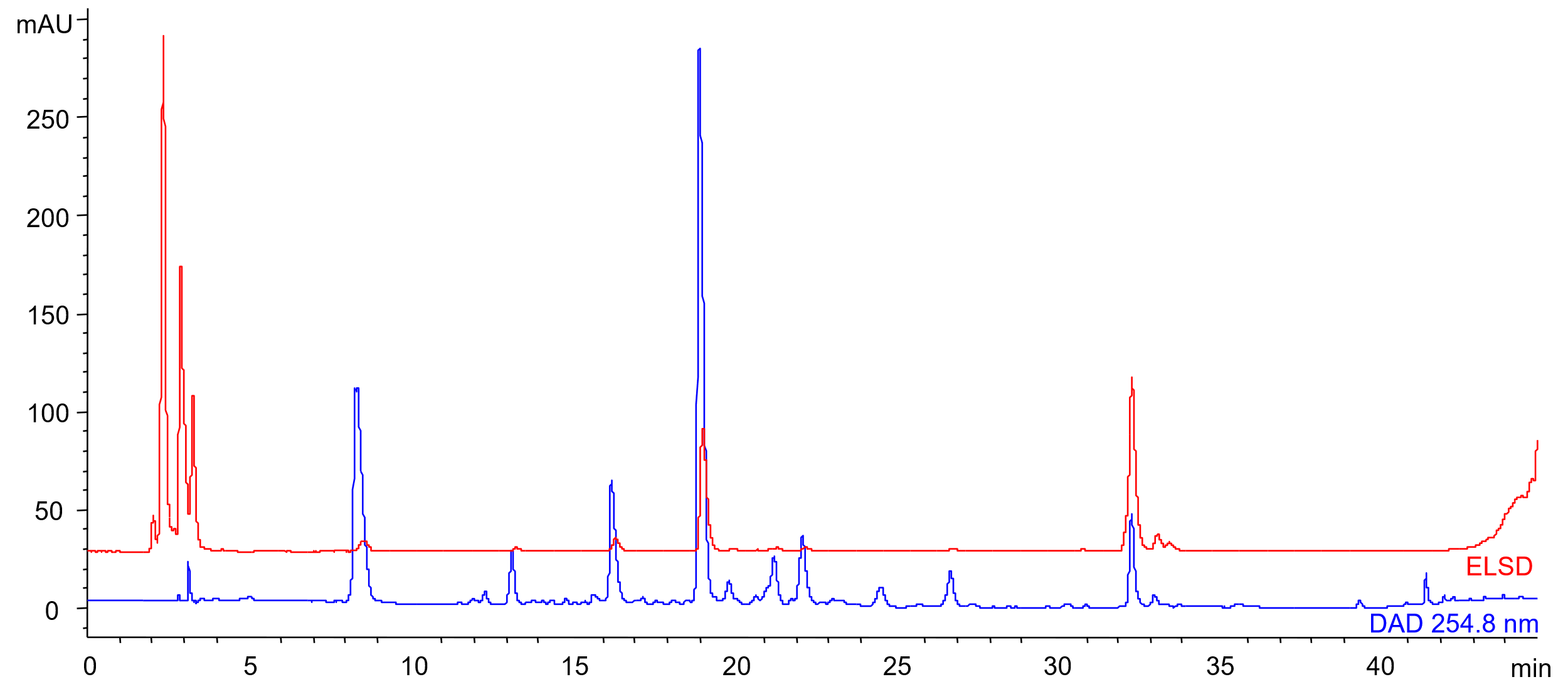


**Figure 7.** HPLC-DAD/ELSD chromatograms of the hydroalcoholic extract of *Clematis vitalba* L.


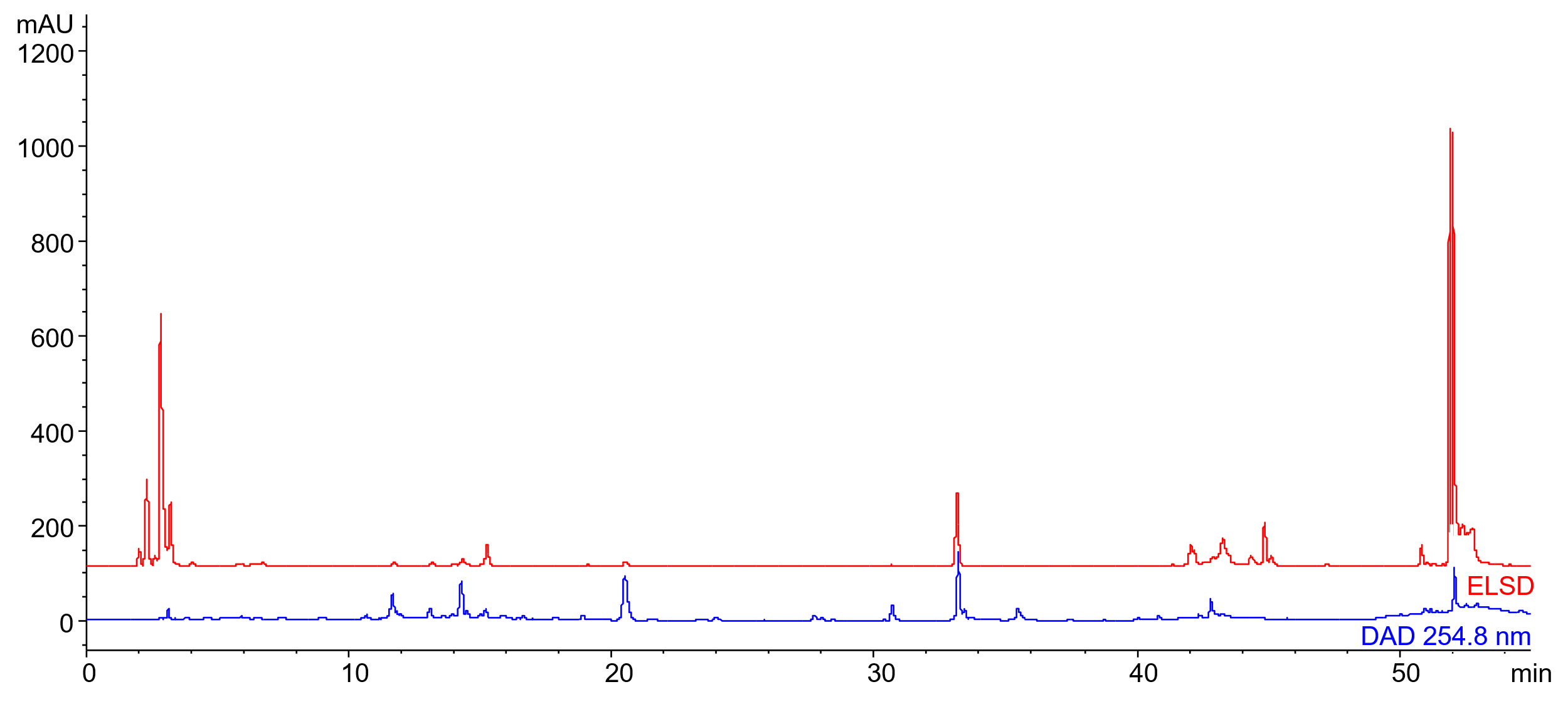


**Figure 8.** HPLC-DAD/ELSD chromatograms of the hydroalcoholic extract of *Cormus domestica* L.


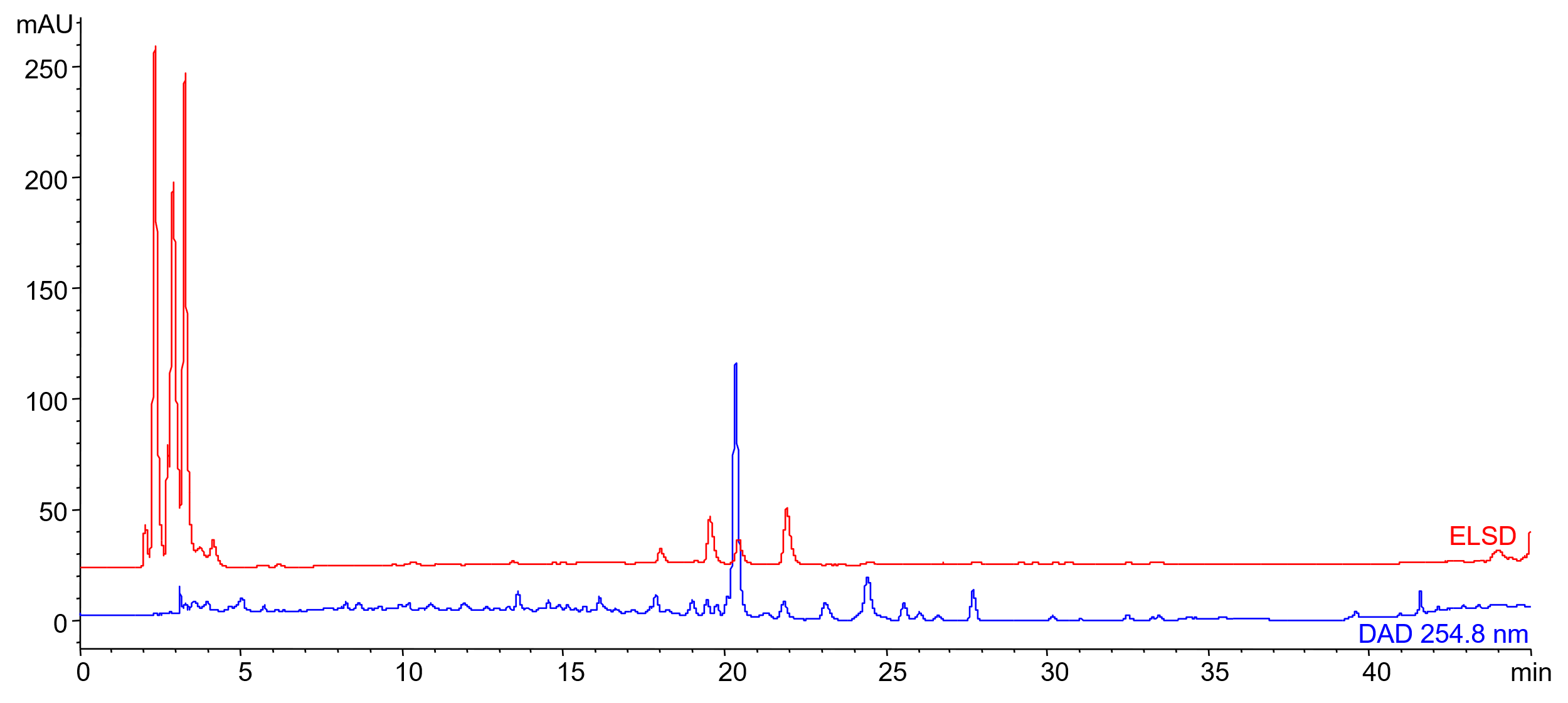


**Figure 9.** HPLC-DAD/ELSD chromatograms of the hydroalcoholic extract of *Corylus avellana* L.


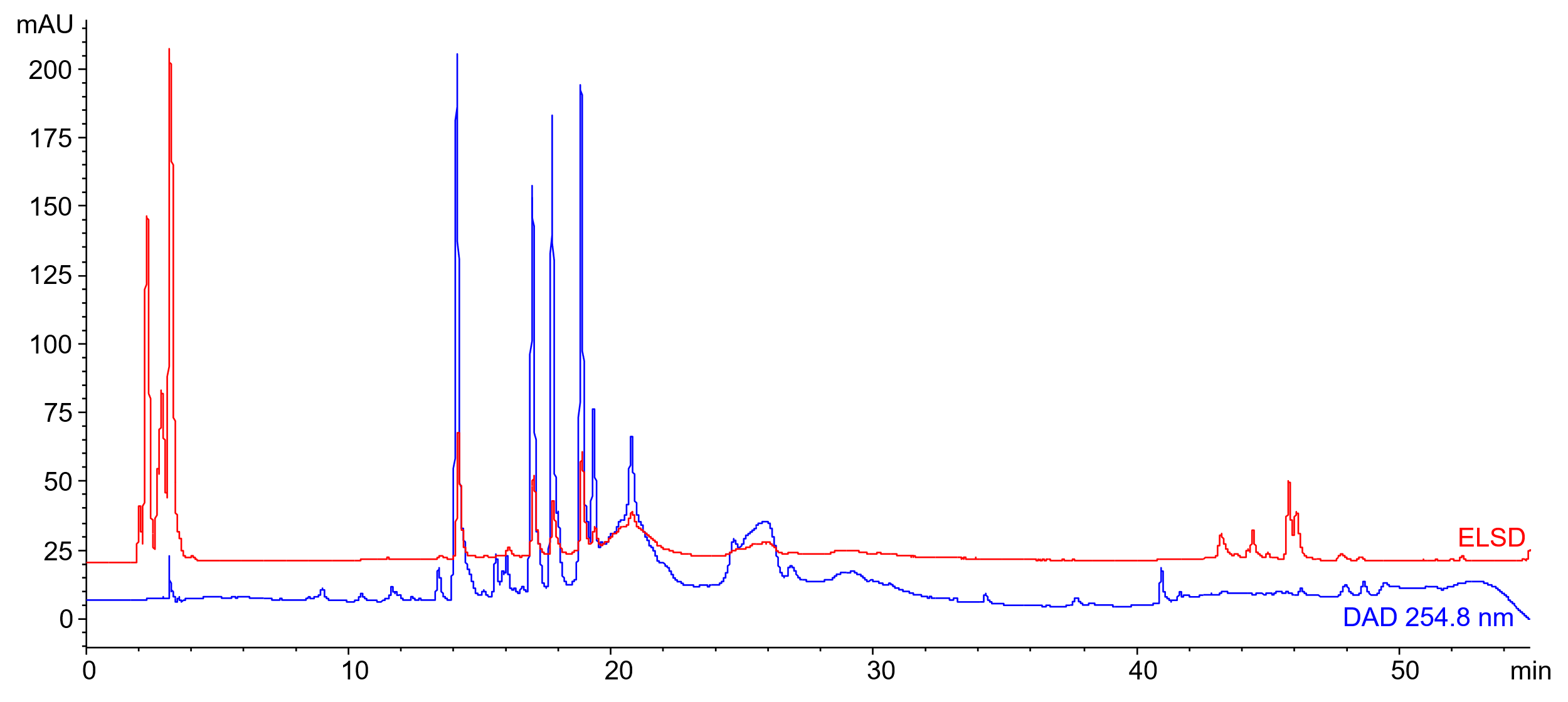


**Figure 10**. HPLC-DAD/ELSD chromatograms of the hydroalcoholic extract of *Dipsacus fullonum* L.


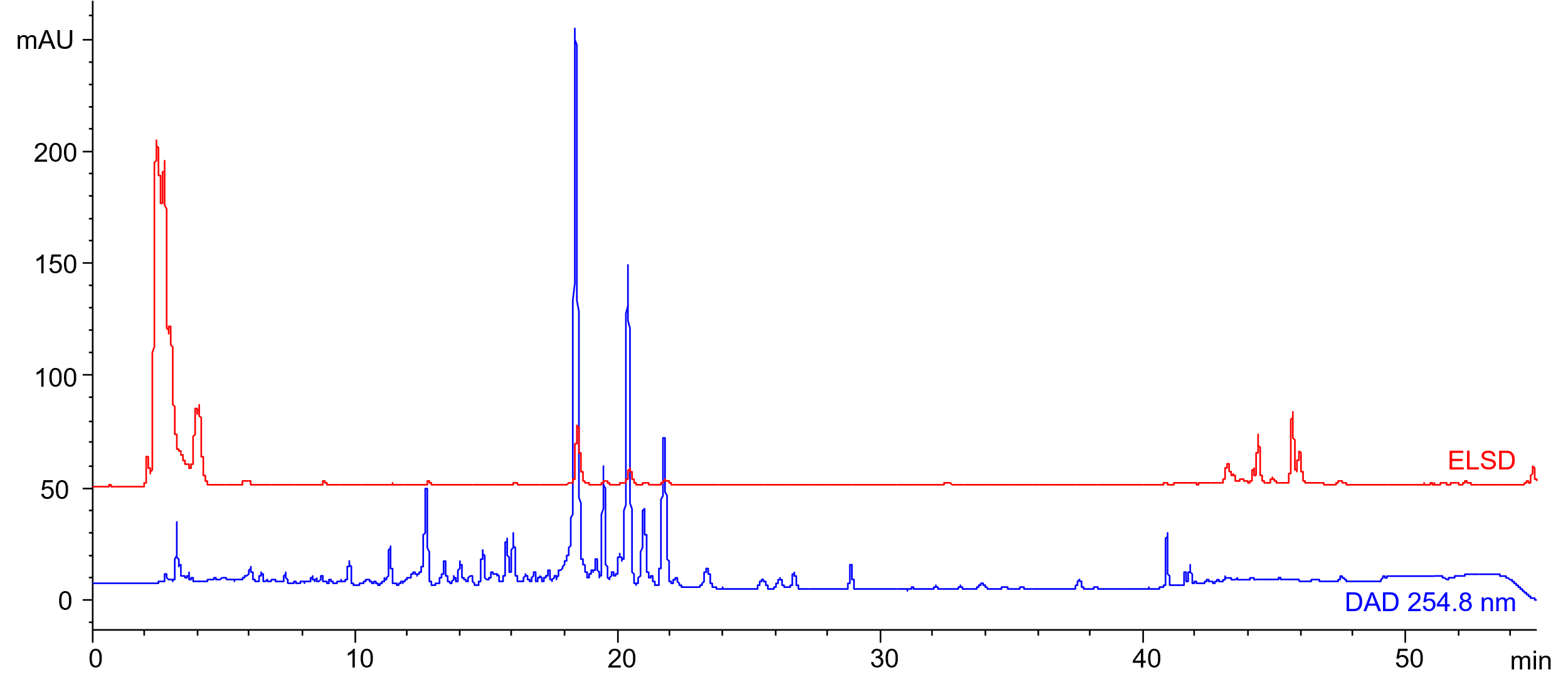


**Figure 11**. HPLC-DAD/ELSD chromatograms of the hydroalcoholic extract of *Lactuca serriola* L.


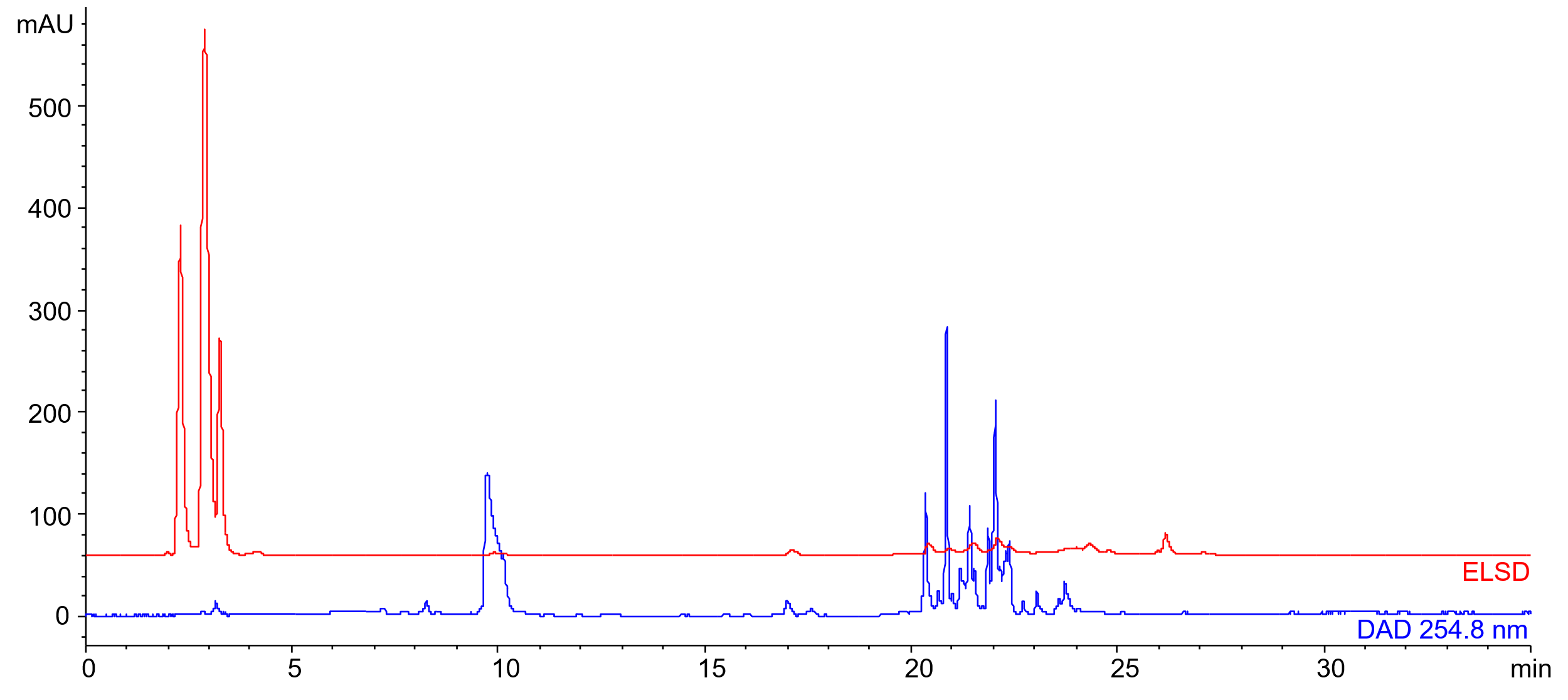


**Figure 12.** HPLC-DAD/ELSD chromatograms of the hydroalcoholic extract of *Narcissus jonquilla* L.


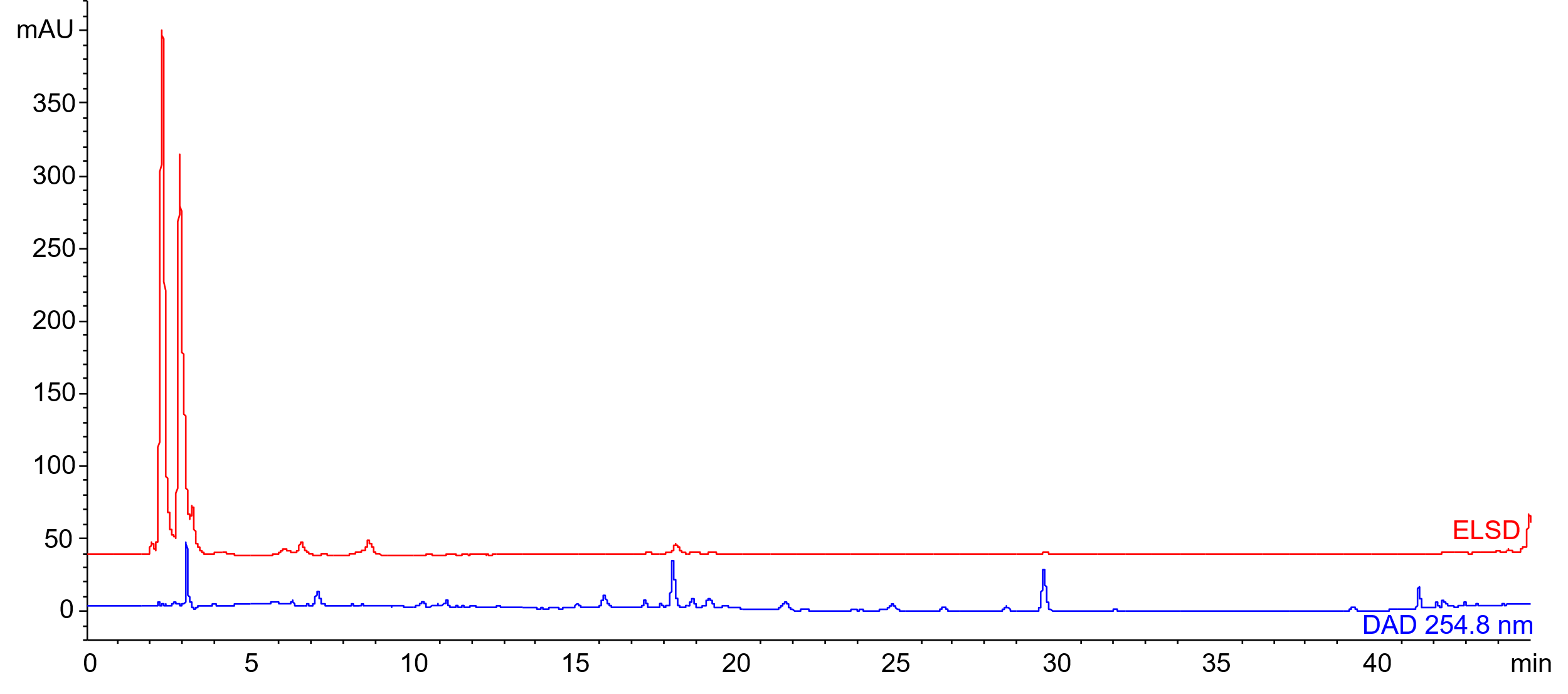


**Figure 13**. HPLC-DAD/ELSD chromatograms of the hydroalcoholic extract of *Plantago lanceolata* L.


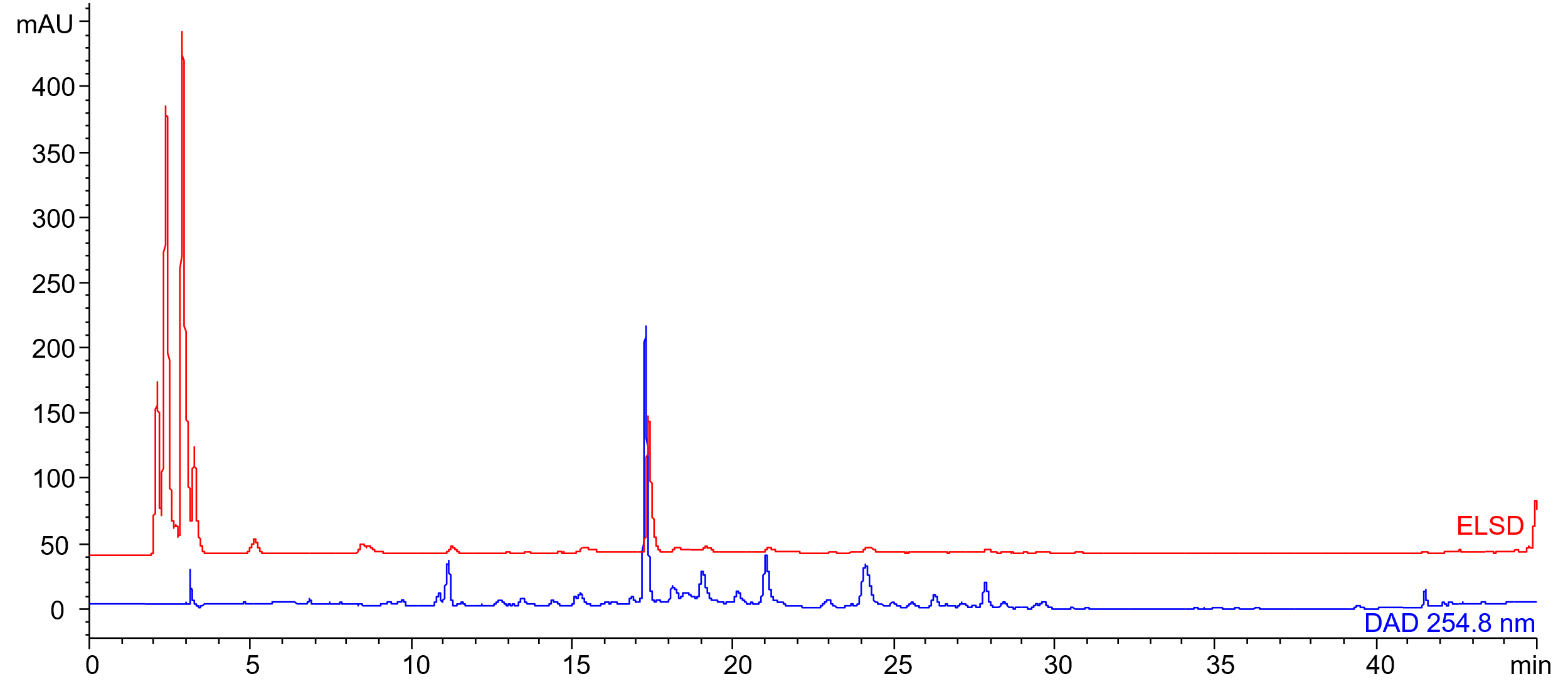


**Figure 14.** HPLC-DAD/ELSD chromatograms of the hydroalcoholic extract of *Plantago major* L.


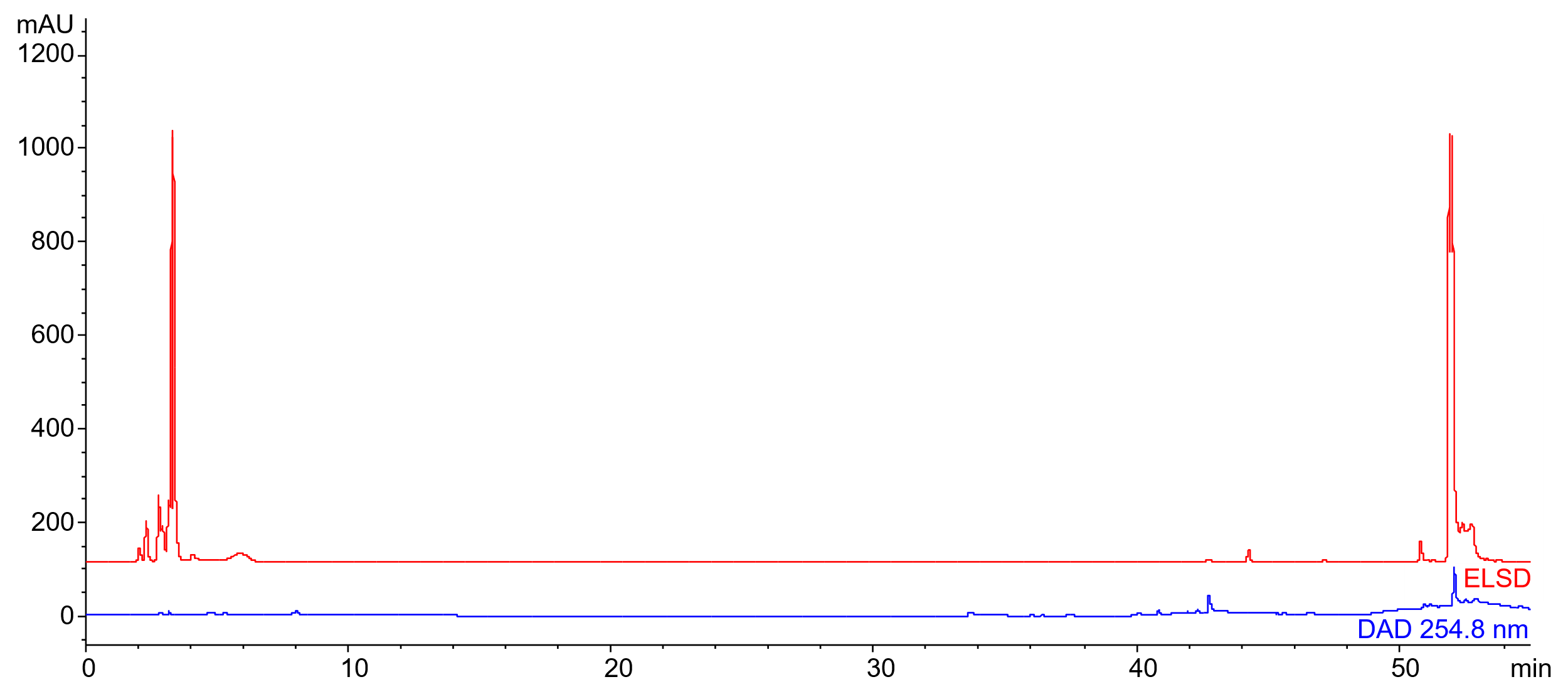


**Figure 15**. HPLC-DAD/ELSD chromatograms of the hydroalcoholic extract of *Polygonatum multiflorum* (L.) All


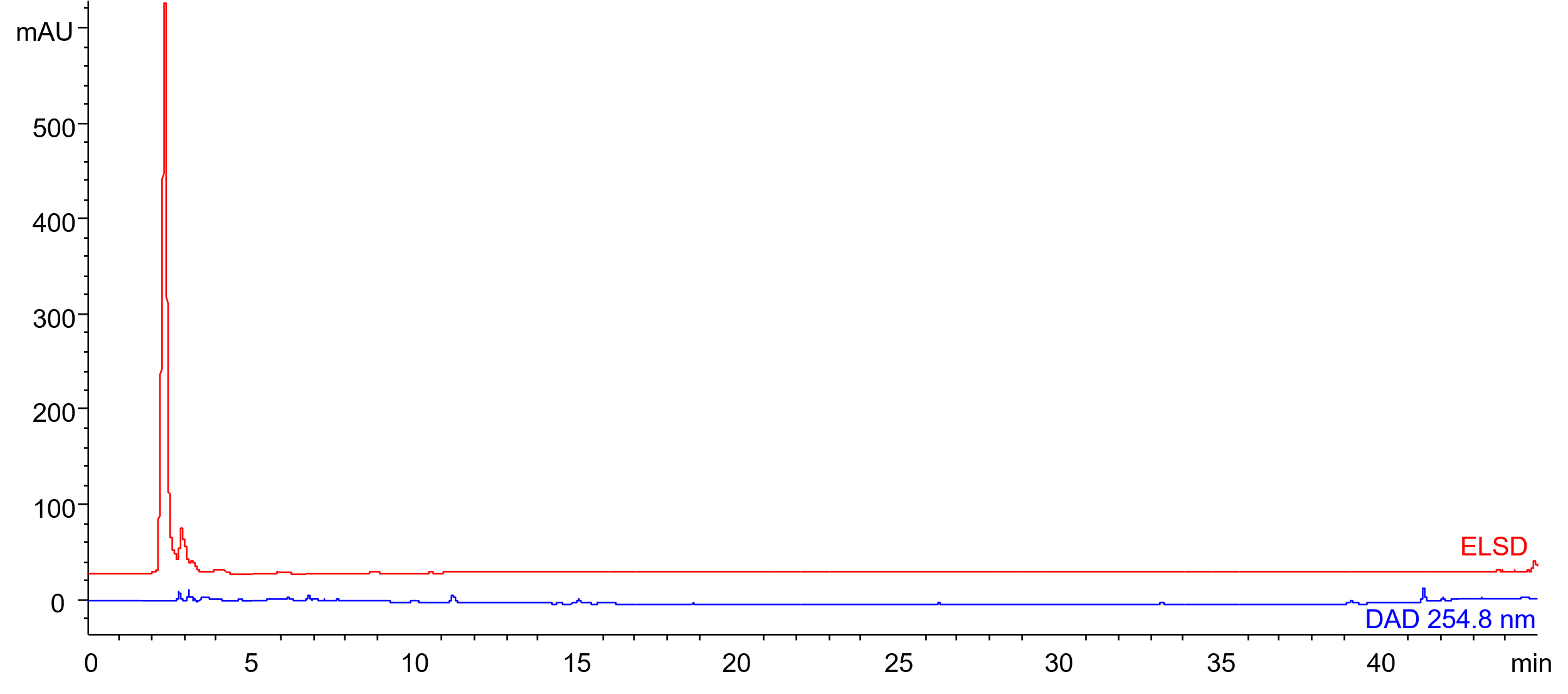


**Figure 16**. HPLC-DAD/ELSD chromatograms of the hydroalcoholic extract of *Portulaca oleracea* L.


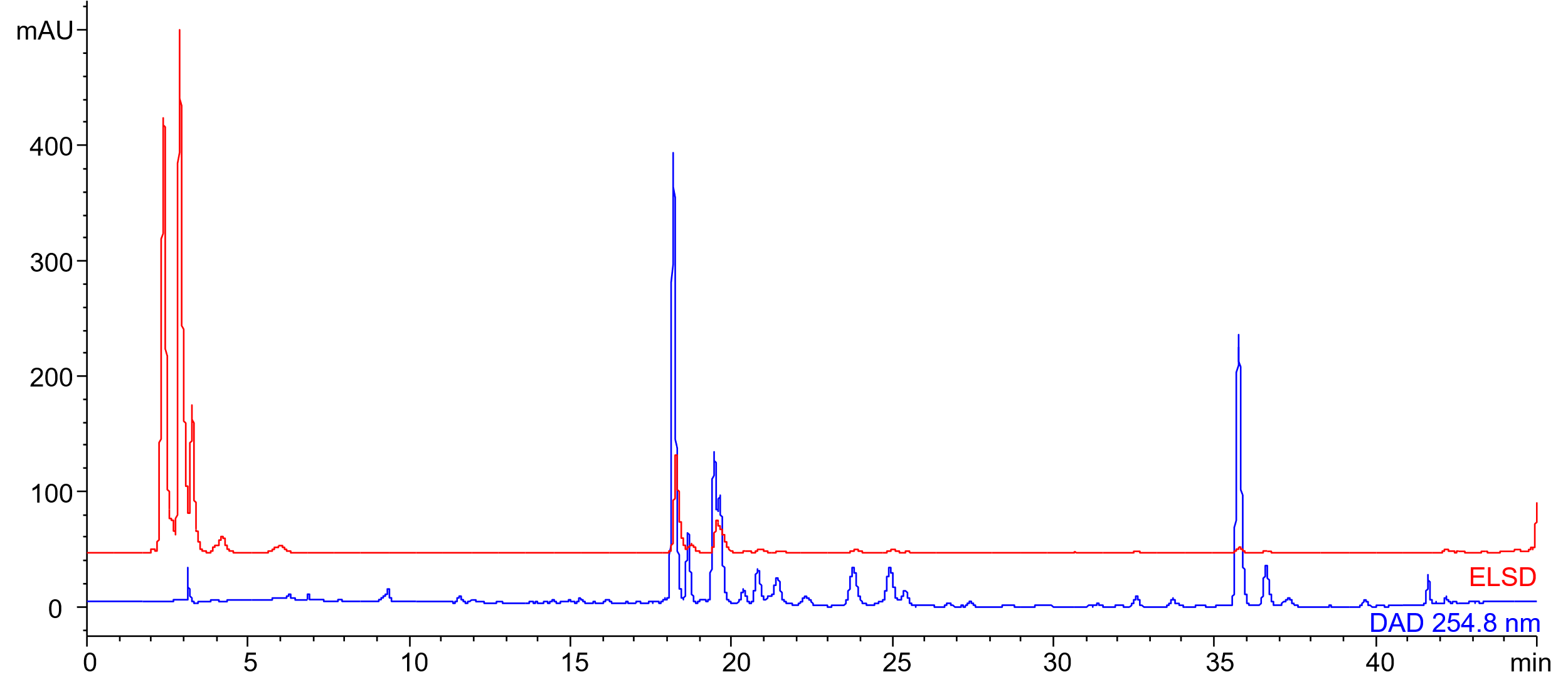


**Figure 17**. HPLC-DAD/ELSD chromatograms of the hydroalcoholic extract of *Primula veris* L.


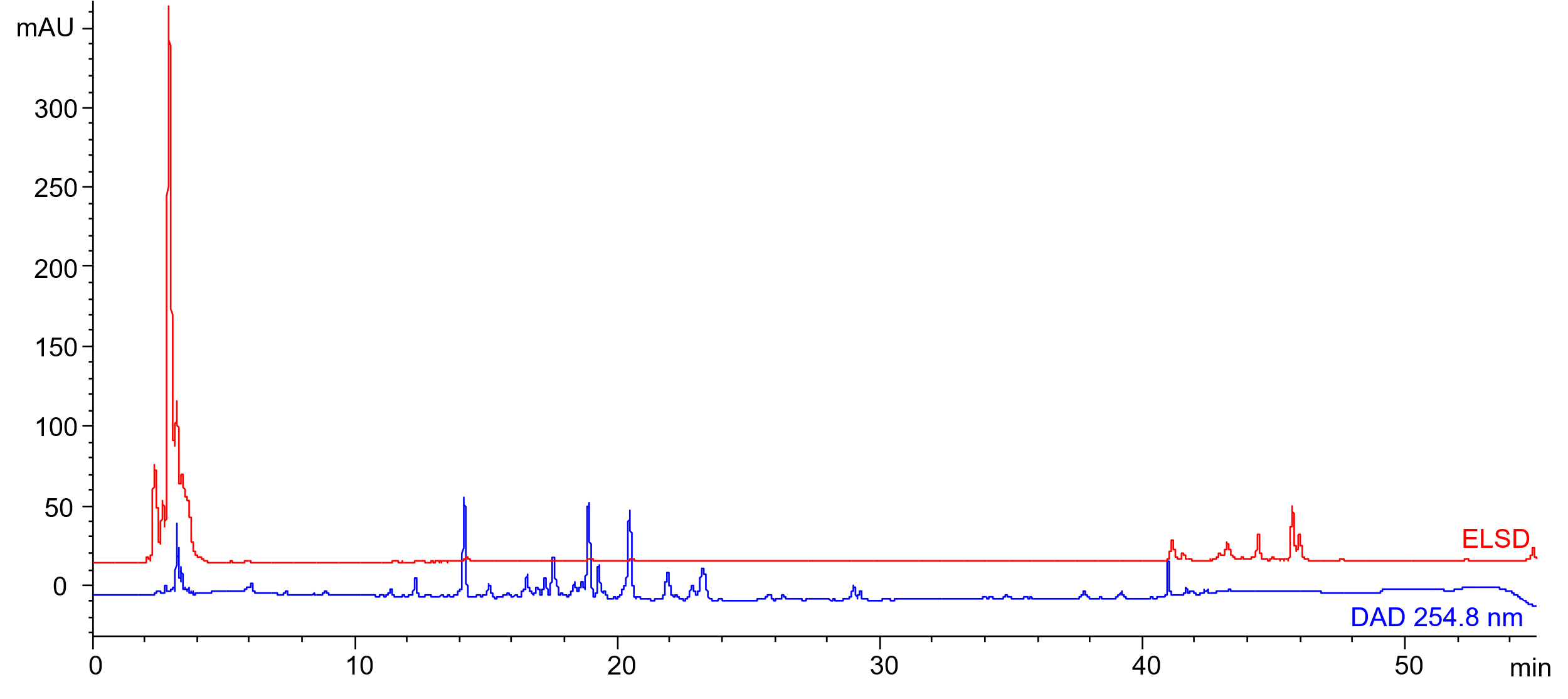


**Figure 18.** HPLC-DAD/ELSD chromatograms of the hydroalcoholic extract of *Spartium junceum* L.

**HPLC-DAD/ELSD chromatograms of *Borago officinalis* L. ethanolic extracts cultivated using rotational indoor farming under different temperature and irrigation conditions**


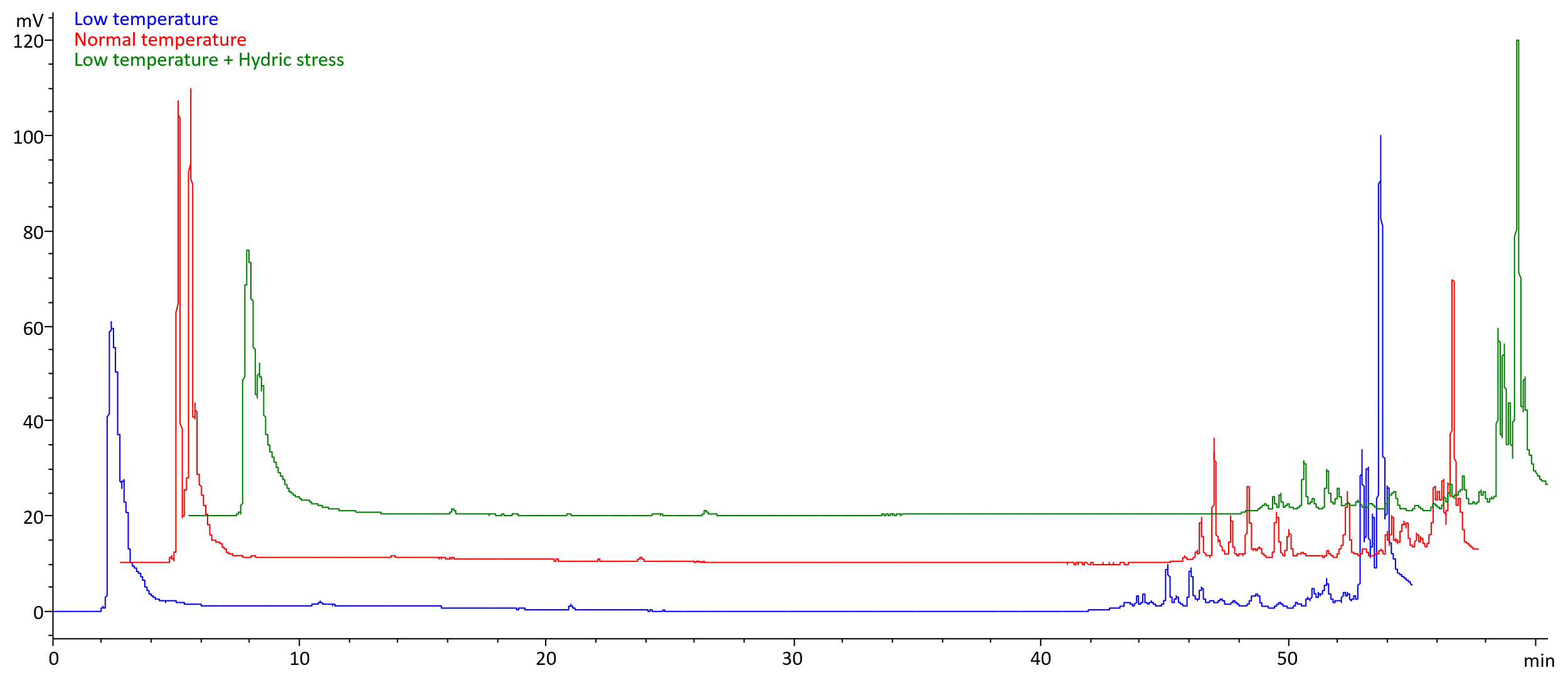


**Figure 19.** HPLC-ELSD chromatograms of the ethanolic extracts of *Borago officinalis* L. aerial parts grown using different conditions


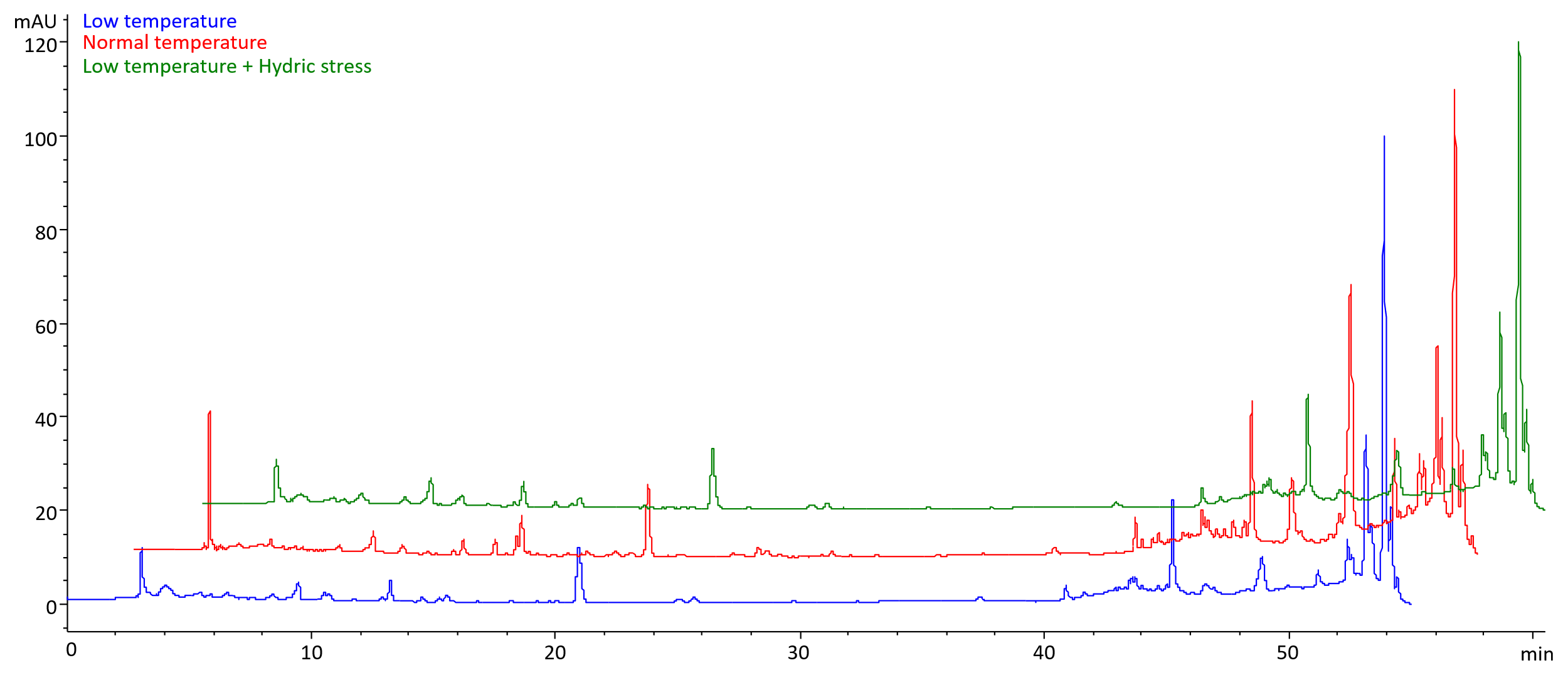


**Figure 20**. HPLC-ELSD chromatograms of the ethanolic extracts of *Borago officinalis* L. aerial parts grown using different conditions
